# Beam Effects in Synchrotron Radiation *Operando* Characterization of Battery Materials: X-Ray Diffraction and Absorption Study of LiNi_0.33_Mn_0.33_Co_0.33_O_2_ and LiFePO_4_ Electrodes

**DOI:** 10.1021/acs.chemmater.4c00597

**Published:** 2024-05-17

**Authors:** Ashley P. Black, Carlos Escudero, François Fauth, Marcus Fehse, Giovanni Agostini, Marine Reynaud, Raphaelle G. Houdeville, Dimitrios Chatzogiannakis, Joseba Orive, Alejandro Ramo-Irurre, Montse Casas-Cabanas, M. Rosa Palacin

**Affiliations:** † Institut de Ciència de Materials de Barcelona, ICMAB-CSIC, Campus de la UAB, Bellaterra 08193, Spain; ‡ ALBA Synchrotron Light Source, Cerdanyola del Vallès 08290, Spain; § Centre for Cooperative Research on Alternative Energies (CIC energiGUNE), Basque Research and Technology Alliance (BRTA), Technology Park, Albert Einstein 48, Vitoria-Gasteiz, Alava 01510, Spain; ∥ IKERBASQUE, Basque Foundation for Science, Plaza Euskadi 5, Bilbao 48009, Spain

## Abstract

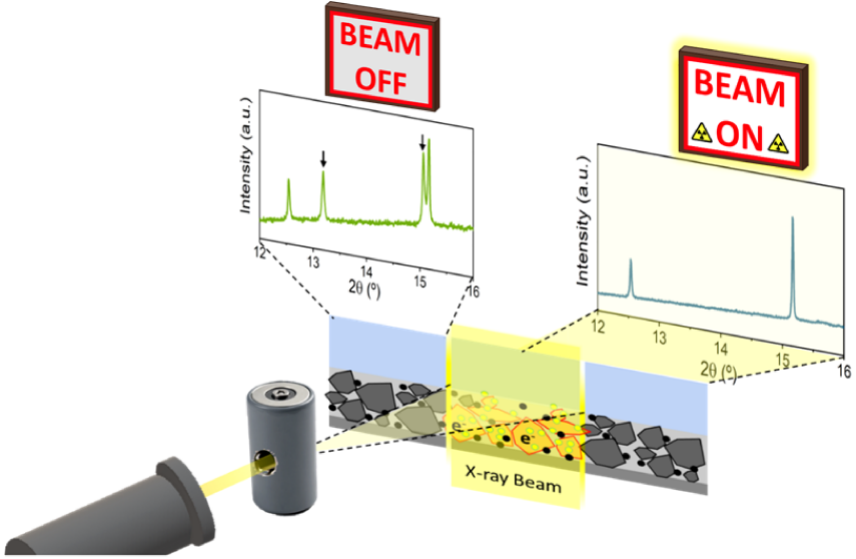

*Operando* synchrotron radiation-based techniques are a precious tool in battery research, as they enable the detection of metastable intermediates and ensure characterization under realistic cycling conditions. However, they do not come exempt of risks. The interaction between synchrotron radiation and samples, particularly within an active electrochemical cell, can induce relevant effects at the irradiated spot, potentially jeopardizing the experiment’s reliability and biasing data interpretation. With the aim of contributing to this ongoing debate, a systematic investigation into these phenomena was carried out by conducting a root cause analysis of beam-induced effects during the *operando* characterization of two of the most commonly employed positive electrode materials in commercial Li-ion batteries: LiNi_0.33_Mn_0.33_Co_0.33_O_2_ and LiFePO_4_. The study spans across diverse experimental conditions involving different cell types and absorption and scattering techniques and seeks to correlate beam effects with factors such as radiation energy, photon flux, exposure time, and other parameters associated with radiation dosage. Finally, it provides a comprehensive set of guidelines and recommendations for assessing and mitigating beam-induced effects that may affect the outcome of battery *operando* experiments.

## 1 Introduction

*Operando* synchrotron radiation-based characterization techniques are becoming a widespread tool, as they allow for nondestructive probing of materials with various depth sensitivities through spectroscopy, scattering, and imaging techniques.^1−4^ Moreover, compared to their laboratory counterparts, they allow for faster acquisition rates, variable penetration depths, and higher spectral or spatial resolution. Synchrotron infrastructures provide access to techniques that require a continuously adjustable source covering an extensive range of photon energies, which enable control over which atomic species or electronic states are probed. In addition, they provide a much higher photon flux than their lab-based counterparts. The use of *operando* techniques has intrinsic advantages, such as the detection of metastable intermediates or studying the dynamics of a kinetic process (see, for instance, pioneering work by Prof. C.N.R. Rao’s group).^5,6^ Moreover, the results are more representative of real operation, and the risk of *ex situ* sample evolution during preparation is avoided. When applied to the battery field, compatibility between the electrochemical cell designs and the experimental setup forces specific design features, and care must be taken to ensure that these do not perturb the electrochemical response of the materials under investigation.^7−9^

However, these techniques do not come exempt of risks. The interaction between synchrotron radiation and samples, particularly within the intricate environment of an operating electrochemical cell, can lead to unforeseen effects in the sample at the exposed area, thereby potentially compromising the experiment’s reliability and biasing data interpretation. While beam-induced effects are well-recognized for their critical impact on characterizing biological samples,^10,11^ macromolecules,^12,13^ soft matter,^14,15^ and even robust inorganic materials,^16−18^ they have, until recently, received scarce attention within the battery research community.

Beam effects range from reversible to permanent alterations, such as loss of resolution in irradiated protein crystals,^11^ beam-induced phase transitions in coordination polymers,^19,20^ changes in crystal nucleation and metal oxidation states,^21,22^ metal ion reduction and water radiolysis in aqueous solutions,^23,24^ and even total sample obliteration in extreme cases, such as with X-ray free-electron lasers (FELs).^25,26^ These effects span a wide range of energies (5–50 keV), techniques, and material types.

The specific mechanisms of such effects may differ depending on how X-rays interact with the sample and originate from secondary events triggered by the energy transferred to the sample upon radiation absorption. Consequently, the dose, expressed in Gy (J kg^–1^) or MGy, which defines the absorbed energy per unit of mass, can be considered a suitable indicator for radiation effects.^11,27^

Recent reports within the battery community have highlighted the impact of prolonged beam exposure on X-ray absorption and X-ray diffraction data collected during *operando* experiments.^28−30^ The reported effects primarily entail a localized hindrance of the electrochemical reaction at the irradiated spot. Remarkably, the data do not indicate signs of degradation, structural changes, or spectral evolution; instead, they reveal a delay in the reactivity, resulting in the observation of an unexpected reaction mechanism. These reports establish a correlation between latency and dose, quantify the extension of the affected area, and suggest a safe radiation dose threshold value.

The mechanism of the beam-induced reaction hindrance is still a matter of debate, possibly having multiple origins that may contribute differently depending on the experimental conditions and the system under investigation. These factors can possibly be thermal heating of the electrode and electrolyte,^14,27^ generation of gas bubbles, degradation of the carbon-binder matrix, resulting in electrical particle disconnection,^31^ or kinetic hindrance induced by secondary electron cascades.^25,27,29^ Understanding and monitoring beam effects during *operando* experiments are thus imperative for obtaining reliable data.

Herein, we present a systematic investigation into these phenomena, conducting a root cause analysis of beam-induced effects during the *operando* characterization of two commercial positive electrode materials used in Li-ion batteries: LiNi_0.33_Mn_0.33_Co_0.33_O_2_ (NMC111) and LiFePO_4_ (LFP). These two materials have been selected as suitable models for studying radiation effects during *operando* experiments because their well-documented lithium intercalation and deintercalation mechanisms provide a clear baseline for data validation, enabling us to easily identify unexpected behaviors by comparing them against previous reports.^32−35^ Also, using these well-known reference materials allowed us to use industrially made laminates with well-defined properties and homogeneity. In addition, they offer the possibility of contrasting the effects between two chemistries that exhibit distinct intercalation mechanisms and voltage profiles. The study spans across diverse experimental conditions involving different electrochemical cell types (coin cells,^36^ Leriche^37^ and LeRiChe’S v2 cells^38^), combined X-ray absorption spectroscopy (XAS), and powder X-ray diffraction (PXRD) techniques and seeks to correlate beam effects with factors such as energy, photon flux, exposure time, and other parameters associated with radiation dosage.

## 2 Experimental Methods

### 2.1 Materials and Cell Setup

Tape casted electrodes containing LiNi_0.33_Mn_0.33_Co_0.33_O_2_ (NMC111) and LiFePO_4_ (LFP) as active materials were purchased from the NEI Corporation (NANOMYTE BE-50E (NMC111); NANOMYTE BE-60E (LFP)). Their compositions in terms of active material:PVDF:Super P mass ratios are (90:5:5) for NMC111 and (88:4:8) for LFP, both casted on a 16 μm thick aluminum foil, with active material loadings of 13.21 and 2.92 mg/cm^2^ for the thick and thin NMC111, respectively, and 7.44 mg/cm^2^ for LFP. 15 mm diameter disks were cut and used as positive electrodes of the *operando* cells, with 16 mm diameter 0.45 mm thick Li disks (MTI Corporation, Richmond, CA, USA) as counter electrodes. Typically, 40 μL of a 1.0 M LiPF_6_ in EC/DMC = 50/50 (v/v) (LP30, Sigma-Aldrich) was used as electrolyte, embedded in a 18 mm diameter glass fiber disk (Whatman, GE Healthcare, 420 μm thick) separator.

Experiments were conducted in three types of electrochemical cells, enabling *operando* testing: Leriche,^37^ LeRiChe’S v2^38^ (both with 200 μm thick Be windows), and modified 2032 coin cells with 75 μm thick Kapton windows.^36^ Cells were assembled in an Ar-filled glovebox and cycled with a BioLogic VSP potentiostat in galvanostatic mode with a potential limitation (GCPL).

### 2.2 *Operando* Measurement Conditions

*Operando* PXRD and combined PXRD/XAS battery experiments were conducted at MSPD^39^ and NOTOS beamlines, respectively, at ALBA Synchrotron (Cerdanyola del Vallès, Spain). The wavelengths used in the PXRD experiments were 0.3535 Å (35 keV) for MSPD and 1.1277 Å (11 keV) for NOTOS. XAS spectra were collected at Mn (6539 eV), Fe (7112 eV), Co (7709 eV), and Ni (8333 eV) K-edges. The beam spot was around 0.2 mm^2^ at NOTOS and 0.5 mm^2^ at MSPD. The photon flux at 11 and 35 keV was estimated to be 1.2 × 10^11^ and 5 × 10^11^ ph/s, at NOTOS and MSPD beamlines, respectively. The beam was monochromatized using a Si (111) double crystal, and harmonic rejection was performed using a silicon mirror at 2.2 mrad. All the XAS spectra were collected in transmission mode employing ion chambers filled with the appropriate mixture of inert gases to absorb around 15% of the photons in the ion chamber before the sample, and around 85% in the ion chamber after the sample and after the reference, since a reference metal foil of the corresponding element was collected at the same time to ensure the energy calibration during the *operando* experiments.

### 2.3 Dose Calculation

The dose, denoting the absorbed energy per unit of mass, was estimated using eq 1, as defined by Blondeau et al.^27^
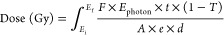
1

Where *F* is the photon flux that reaches the electrode (ph/s), *E*_photon_ is the energy of the photon (eV), *t* is the exposure time (s), *T* is the transmission of the absorber at a given energy, and at the denominator, the mass is calculated as the area irradiated by the beam (*A*) times the electrode thickness (*e*) and the absorber density (*d*). In order to estimate the photon flux that reaches the electrode, a 10 μm thick Si transmissive photodiode^40^ was positioned in the sample position to directly measure how the flux was reaching the electrochemical cell, considering all the elements in the beamline optics path (including attenuators just before the sample, if used). The absorption of all elements present within the electrochemical cell prior to reaching the sample (positive electrode material) was also taken into consideration. The absorption (1 – *T*) of the electrode material, the aluminum attenuators, the (Be/kapton) windows, the separator, and the electrolyte were estimated with XOP software,^41^ and further details are given in the Supporting Information. For the XAS experiments, the flux at each increment of energy of 25 eV was considered, as it can change more than 2 orders of magnitude depending on the beamline design. The dose, expressed in MGy, was estimated for each measurement (PXRD and XAS). The time-dependent dose is calculated by adding the dose of successive measurements and is displayed on the vertical right axis of the PXRD diffractogram or XANES spectra throughout this study. The estimated dose values for each measurement are given in Table S1.

### 2.4 Measurement Protocol

For all experiments, except for those reported on Section 3.1 and Section 3.5, data acquisition was conducted following a protocol that allowed to measure four cells sequentially maintaining the same time interval between consecutive measurements on the same cell (23 min) while proceeding with the electrochemical testing uninterruptedly. Typically, the cycling rate was kept between C/7 and C/10, depending on the specific capacity of the tested material, which allows the evolution of the structural and spectral changes to be followed with acceptable time resolution within the acquisition time constraints imposed by the beamline instrumentation. In order to decouple the effect of the PXRD and the XAS measurements, which are acquired at significantly different energies, two positions A and B were measured in each cell, with PXRD being performed at both spots and XAS only acquired at spot B. The acquisition time for the PXRD at 11 keV was set at 1 min, and the acquisition of XAS spectra was fixed to 1 min for XANES and 3 min when EXAFS was collected. Details on cell configuration and measurement protocols are given in Figure S1Table S1. In addition, in an attempt to avoid total beam-induced reactivity inhibition, for the experiments reported in Sections 3.2, 3.4, 3.6, and 3.7, aluminum attenuators were introduced just before the samples, which result in a photon flux reduction of 70 to 90% of the flux at Ni K-edge for 100 or 250 μm of Al, or a reduction of 49 to 81% of photon flux with 100 or 250 μm Al at 11 keV.

## 3 Results

In order to better assess the results achieved, it is useful to recall the expected behavior of the electrode materials selected for this study. NMC111 features an average nominal voltage of 3.75 V vs Li/Li^+^and capacities exceeding 150 mAh/g when cycled between 2.7 and 4.3 V. It exhibits a sloping voltage vs capacity profile, and the lattice parameters contract and expand as the lithium content changes. Its redox mechanism involves a complex interplay between solid solution and biphasic reactions, which is influenced by factors such as the composition, charging rate, and temperature. Solid solution predominates, especially at the initial and final stages of charge and discharge, while biphasic regions occur during at intermediate redox stages where increased cation disorder and lattice strain can trigger phase transitions.^33^ The corresponding changes in the X-ray absorption spectroscopy (XAS) spectra are primarily characterized by a significant continuous shift of the Ni K-edge, as Ni is the main redox active element, whereas only minor changes in the K-edge XANES spectra of Mn and Co are observed that correspond to changes in their ligand field induced by the oxidation/reduction of Ni.^35,42^ However, LFP reacts following a two-phase transition process involving two isostructural triphylite-type phases, LiFePO_4_ and FePO_4_ (FP), which present different lattice parameters. In the course of lithiation/delithiation, the intensity of the diffraction peaks of one phase decreases to the expense of those belonging to the other, as the later nucleates and grows.^34^ Accordingly, the voltage vs capacity profile of this system presents a characteristic flat plateau. The corresponding changes in the XAS spectra are primarily characterized by a significant continuous shift of the Fe K-edge.^43^ LFP has capacities close to 170 mAh/g when cycled between 2.5 and 4.1 V vs Li/Li^+^, with a nominal voltage of 3.45 V vs Li/Li^+^.

### 3.1 Total Reactivity Inhibition

**Figure 1:**
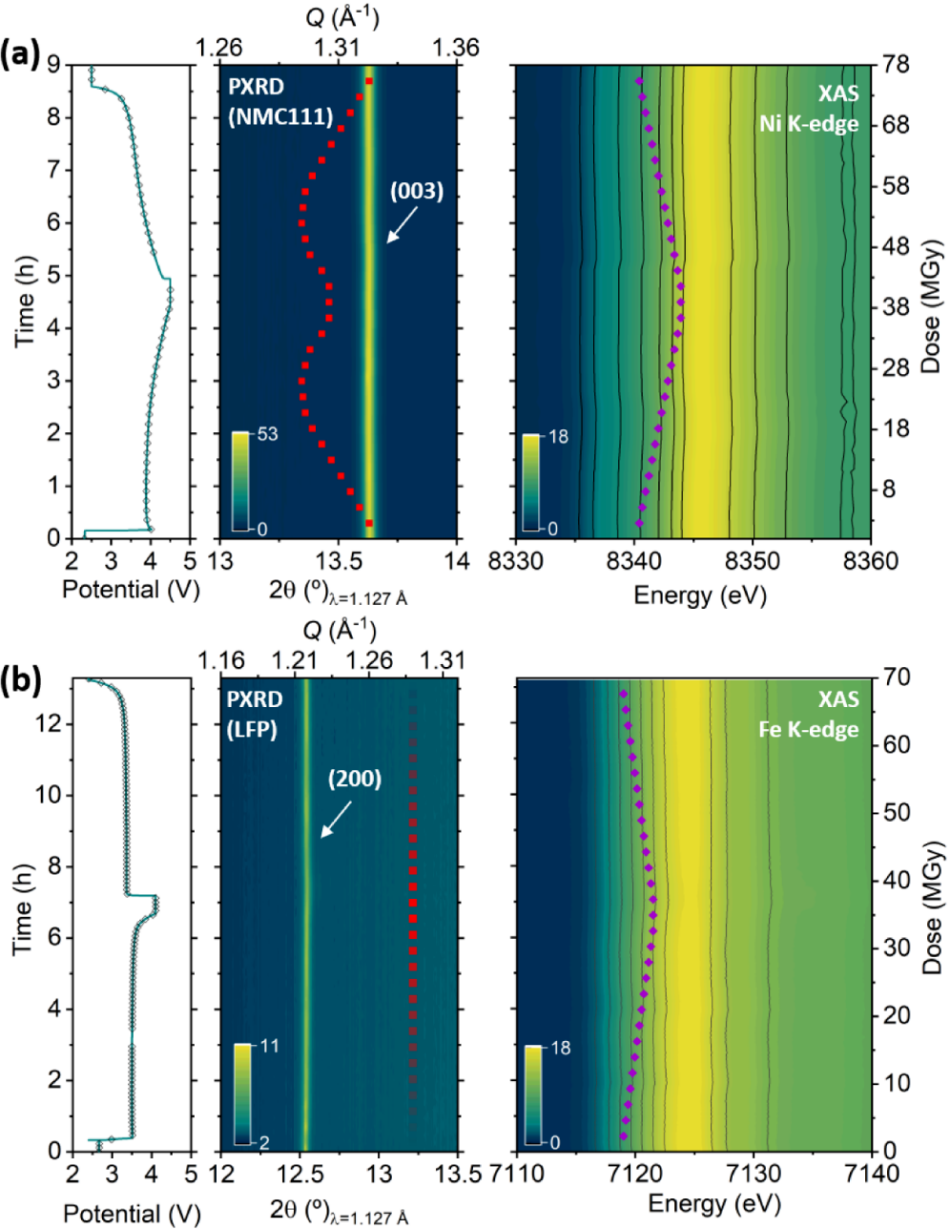
*Operando* X-ray diffraction pattern (center), X-ray absorption spectra measured (right), and electrochemical curve (left) for NMC111 (a) and LFP (b) electrodes measured in Kapton window coin cells. Dots marked in the electrochemical curve indicate measurement points. Red and purple dotted lines indicate expected evolution in the absence of beam-induced effects.

A first combined XAS/XRD *operando* experiment was carried out with a straightforward and simple data acquisition protocol. The cells were cycled in galvanostatic mode, while they were continuously exposed to the synchrotron X-ray beam for the sequential acquisition of the PXRD patterns and XAS spectra. For each measurement, a diffraction pattern and EXAFS spectra of all three transition metal K-edges for NMC111 or Fe for LFP were collected at the same position. The electrochemical profiles of both materials, displayed in Figure 1, show the expected voltage profile and capacities, i.e., 187 mAh/g, 2.5–4.5 V@C/10 for NMC111 and 160 mAh/g, 2.5–4.1 V@C/10 for LFP. While this indicates that the active material in the electrodes has undergone the expected oxidation and reduction reactions, no relevant changes are observed in neither the diffraction patterns nor the absorption spectra of NMC111 or LFP. Stack plots of extended sections of the diffractograms are available in the Supporting Information (Figure S2) together with their corresponding XANES spectra.

**Figure 2:**
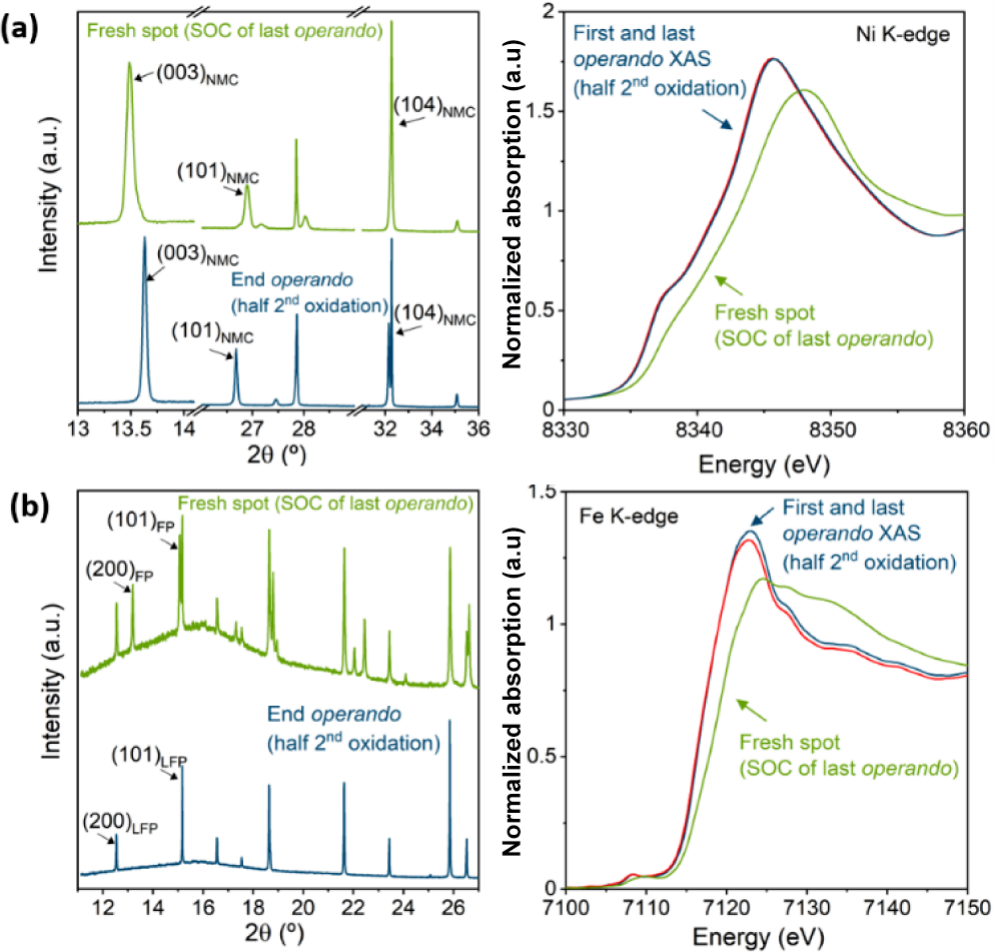
Last *operando* X-ray diffraction pattern (left) and X-ray absorption spectra (right) and corresponding data at a nonirradiated (fresh) spot at the same state of charge (half 2nd oxidation) of NMC111 (a) and LFP (b) electrodes.

In view of these puzzling results, the *operando* measurement was interrupted at about half way through the second oxidation, and multiple locations within the electrode that had not been previously exposed to the beam were examined (see Figure 2). The diffraction patterns and absorption spectra at these unexposed spots were found to exhibit the expected structural and spectral changes with respect to the pristine state. It is noteworthy that, other than the apparent total lack of reactivity at the spots under continuous synchrotron light irradiation, the data revealed no signs of degradation or chemical or structural alterations. This apparent lack of reactivity seemed to be associated with radiation exposure. Since the proportion of the electrode area subjected to irradiation is as little as 0.1%, its contribution to the overall experimental capacity is negligible, and any reaction inhibition at that point could not be inferred from the electrochemical behavior. Hindrance in reactivity induced by radiation has recently been reported in *operando* PXRD experiments conducted on standard graphite vs LiNi_0.6_Mn_0.2_Co_0.2_O_2_ (NMC622) or LiNi_0.8_Mn_0.1_Co_0.1_O_2_ (NMC811) pouch cells^29^ as well as for LFP in AMPIX cells.^28^ To assess the beam effects, the time-dependent dose was calculated according to eq 1, as detailed in the Experimental Methods section, and is displayed on the vertical right axis of the XANES spectra of Figure 1. The dose rate imposed was >5 MGy/h with an uninterrupted exposure to the beam, so that the total dose after a charge and discharge cycle was >70 MGy. Jousseaume et al.^29^ reported a clear kinetic limitation in NMCs for 11 MGy, which is in agreement with our findings (total lack of reactivity at the measured spot for doses >70 MGy).

### 3.2 Influence of Cell Type

The lack of electrode reactivity observed during *in situ* and *operando* experiments has often been attributed to deficient functioning of the electrochemical cell, lack of electrical contact, or stack pressure induced by the geometrical constraints (e.g., flexibility of the window) at the measurement spot.^44,45^ This is particularly critical for Kapton window coin cells, owing to the lack of rigidity and electrical conductivity of the Kapton window itself. Since we observed absence of reactivity at the irradiated spot also when using Leriche-type cells with rigid and conductive beryllium windows employing similar levels of radiation dose (Figure S1), a comparative study was conducted using three different cell setup types. In the case of the LeRiChe’S v2 cell, the unused space inside the cell is minimized, and both electrodes are tightly enclosed between two beryllium windows, which ensures an even pressure across the cell stack.

**Figure 3:**
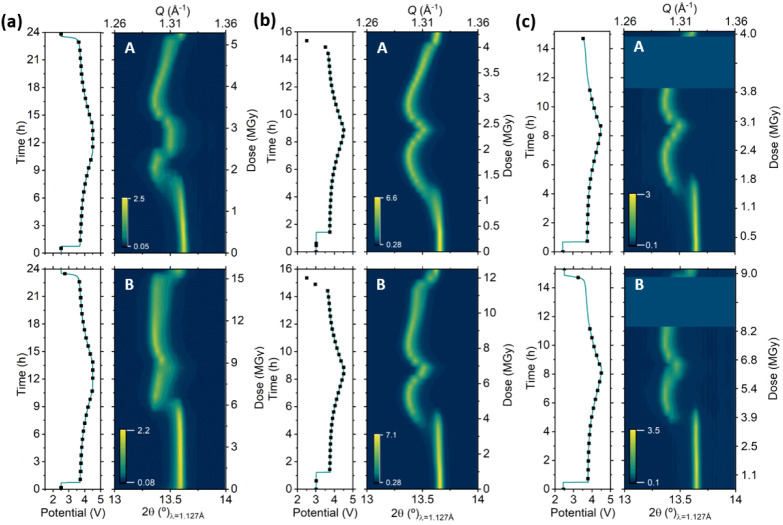
*Operando* X-ray diffraction patterns (zoom in on reflection 003) for NMC111 in spots A (up) and B (down) and corresponding electrochemical curves acquired in similar conditions in Leriche 1.0 cells (a), hexagonal LeRiChe’S v2 cells (b), and Kapton window coin cells (c). Dots marked in the electrochemical curve indicate measurement points.

Figure 3 displays the contour plot of the (003) diffraction peak of NMC111 as a function of time in modified coin cells, Leriche 1.0 cells, and LeRiChe’S v2 cells. Data acquisition was conducted following the protocol detailed in the experimental section, where each cell was measured at two positions A and B, with PXRD being performed at both spots and XAS only acquired at spot B. The dose rate was fixed by imposing 23 min between consecutive measurements, and photon flux was reduced by placing 100 μm Al just before the sample. The dose estimation for each cell, displayed on the vertical right axis of the PXRD diffractogram, reflects the slight differences in the measurement conditions, such as the number of edges or energy range sampled on each experiment. Despite minor differences in the experimental conditions (potentiostatic voltage hold of 1 h at the end of each half cycle for the experiment in Leriche 1.0 cell, beam lost for 4 h during the coin cell test), relevant conclusions can be driven from these comparative experiments. The first is the different reactivities observed at the A and B spots for all three *operando* cell types. A delay in the peak displacement of the NMC (003) reflection is observed in all cases. The time fraction of the cycle during which the hindrance is more severe appears to be proportional to the dose. Since the dose is substantially higher for the B spot than for the A spot (the latter involves XAS in addition to PXRD), the delay or lack of peak evolution at the B spot is more pronounced. The peak position and intensity of the NMC (003) reflection remain constant for a significant period of time during oxidation, and changes only appear above 4 V. Beyond this voltage threshold value, the peak position shifts quickly to lower angles, which would suggest an abrupt two-phase transition at the measurement spot. For the experiment conducted in LeRiChe’S v2 cells (Figure 3b, B point), where the dose per cycle is slightly lower than for the Leriche 1.0, the delay or hindrance seems to be momentarily overcome after this abrupt phase transition as for the remainder of the first charge the (003) reflection appears to evolve smoothly according to the overall state of charge of the cell. In contrast, the Leriche 1.0 cell continues to exhibit signs of hindrance in the peak shift, persisting beyond the abrupt phase transition. Following with the cell LeRiChe’S v2, the peak evolution at spot B shows low signs of hindrance during the first half of the discharge cycle, compared to that deduced from the measurements at spot A, revealing an apparent recovery of reactivity. The peak shift is however clearly hindered for the last half of the discharge, below 4 V, the region in which the voltage profile exhibits a less pronounced decline. However, the peak evolution that the Leriche 1.0 and coin cells exhibited at spot B shows indisputable evidence of a strong degree of hindrance throughout the entire cell discharge. From these experiments, we can conclude that at dose levels below 20 MGy, the inhibition is not total and that different levels of apparent reactivity are observed for NMC111 in all three cell types tested. The degree of hindrance is proportional to the dose, as can be inferred by comparing the evolution of NMC (003) reflections at spots A and B, upon both oxidation and reduction. Coincidentally or not, the delay in reactivity seems to be more evident in the regions in which the voltage profile is flatter, especially below 4 V. This correlation between recovered reactivity and slope of the curve is evidenced, for all cells, with an abrupt change in the NMC (003) reflection at the very end of the reduction.

Comparative studies aiming to assess the cell influence on the apparent beam-induced effects on LFP electrodes were also conducted in modified coin cells and Leriche 1.0 cells. The contour and stack plots for the (200) diffraction peaks of LFP and FP as a function of time and radiation dose are shown in Figure S4. In the case of LFP, apparent reactivity is recovered in both cell setups when the dose is reduced from 70 to below 20 MGy per electrochemical cycle (Figure 1b). For this system, even at these intermediate dose levels, the reaction is hindered charge/discharge processes as the intensity of the LFP or FP peak remains unchanged during most of the redox reaction. The phase transformation is only observed at the very end of the charge/discharge where the steep slope in voltage seems to trigger an abrupt phase transformation. Beam-induced hindered reactivity on LFP electrodes was also reported by Christensen et al.^28^ Overall, and similarly to NMC111 at intermediate dose levels (<20MGy/cycle), the apparent hindrance is only partially or abruptly overcome when the electrode is subjected to rapid changes in voltage.

In conclusion, the effect of uniform pressure in the cell with respect to the observation of beam-induced effects seems to be minor, if any, as these are mostly correlated to the dose.

### 3.3 Influence of the Absorption Edge Energy: Element Specificity

**Figure 4:**
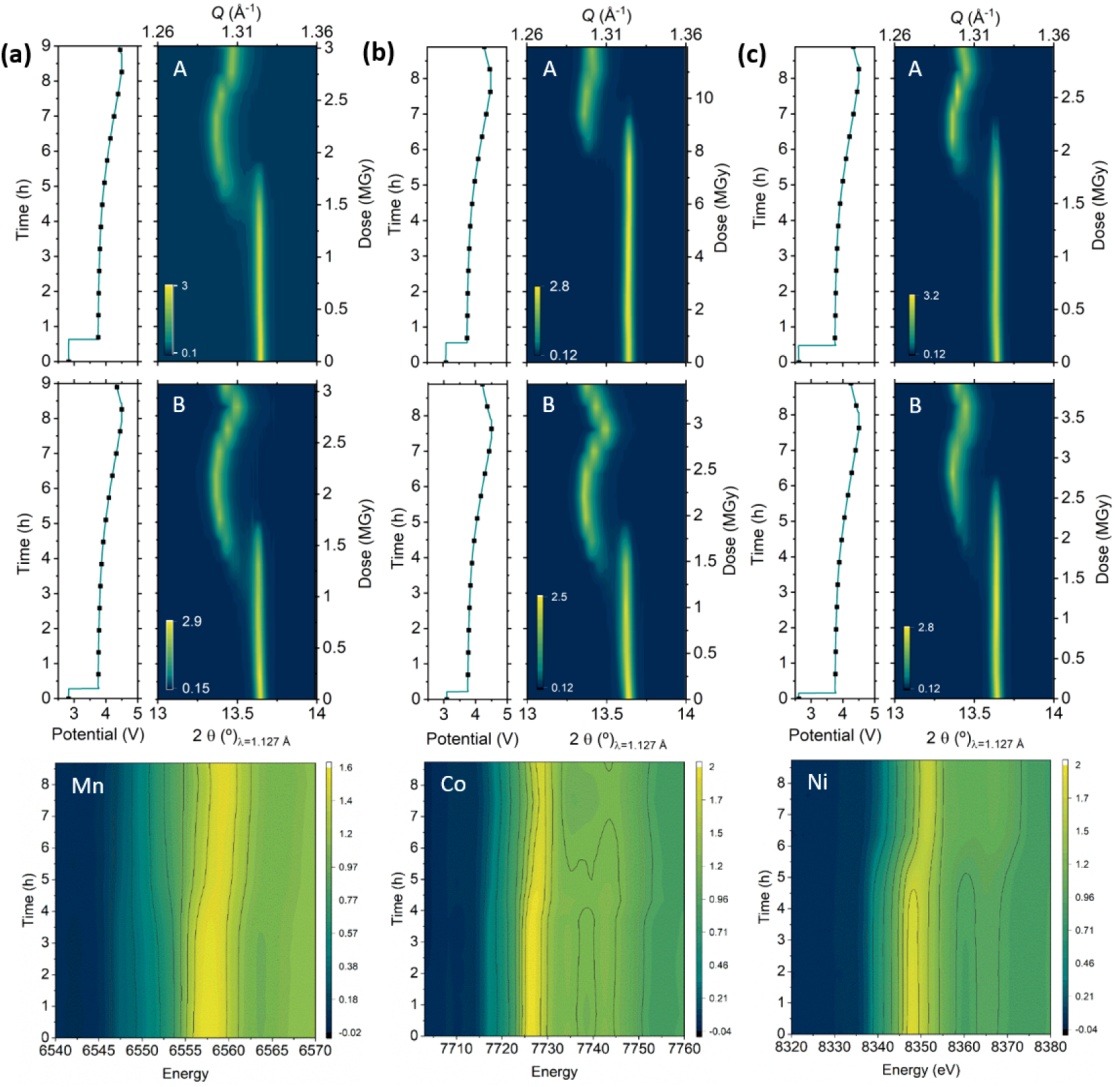
*Operando* X-ray diffraction patterns (zoom in on reflection 003) for NMC111 at spots A (top) and B (center), together with the corresponding X-ray absorption spectra measured only at spot B (bottom) and corresponding electrochemical curves acquired using Kapton window coin cells under different measurement conditions. Exposition time to 11 keV radiation was 4 times longer per measurement at spot A of cell (b) than for cells (a) and (c). At spot B, only K-edge spectra of Mn, Co, and Ni were, respectively, measured on cells (a), (b), and (c). Dots marked in the electrochemical curve indicate measurement points. Stack plots of the time-dependent XRD and XAS of these same samples are displayed in Figure S6.

In order to further characterize the radiation effects at different dose and absorption edge energies, three NMC111 coin cells were measured in a combined XAS and PXRD experiment following the two-spot measurement protocol previously described, where PXRD was measured at spots A and B, and for this experiment, only one edge (Mn, Co, or Ni) XAS spectra were also measured for each cell at the B spot. Since the experiment aimed at correlating possible element specific driven contributions to the observed beam effects, the acquisition time of all three XAS spectra was fixed to 60 s. In addition, and for the sake of comparison, spot A was irradiated for three additional minutes at 11 keV for one of the cells (Figure 4b). The results of this experiment are depicted in Figure 4 and again allow to observe differences in the dynamics of the lithiation mechanism that correlate with the dose. Spots A, where only PXRD was measured at 11 keV, show strong hindrance or delay in the phase evolution, with no appreciable changes in (001) reflection until the voltage changes from 4.0 to 4.1 V for cells (a) and (c), where the cumulative dose was kept below 3 MGy for a half cycle. Cell (b) with cumulative dose above 10 MGy per half cycle presents a stronger inhibition with no signs of reactivity until the voltage increases from 4.2 to 4.3 V. Surprisingly, for spots B of cells (a) and (c), despite being additionally irradiated at the absorption edges, the dose does not increase significantly when compared to their respective spots A, where only 1 min of PXRD was taken. This is mostly due to the changes in photon flux throughout the energy window of NOTOS beamline in this configuration (see Figure S5), where the flux increases from 4 × 10^10^ to 1.2 × 10^11^ from Mn K-edge to 11 keV. As a consequence, the contribution of a 60 s XANES acquisition at the K-edge energy of Mn is only 0.1 MGy/cycle. No significant differences in reactivity hindrance can thus be appreciated between measurements performed at different edge energies (Mn, Co, and Ni) except for a slightly stronger inhibition for the Ni K-edge. This is likely related to the higher photon flux, which results in a higher dose. Beam-induced effects at spots A do not seem to show direct effect on measurements at spot B. This is particularly salient for cell (b) for which hindrance at spot B is comparable to spot B of cell (a), despite having a strongly inhibited spot A. These observations indicate that the area of the electrode affected by the radiation is roughly constrained to the beam size or, at least, smaller than the distance between A and B spots (3 mm). The contour plots of the XAS spectra of Mn, Co, and Ni show a direct correlation between the inhibition observed for PXRD and XAS.

### 3.4 Influence of Photon Flux and Exposure Time

**Figure 5:**
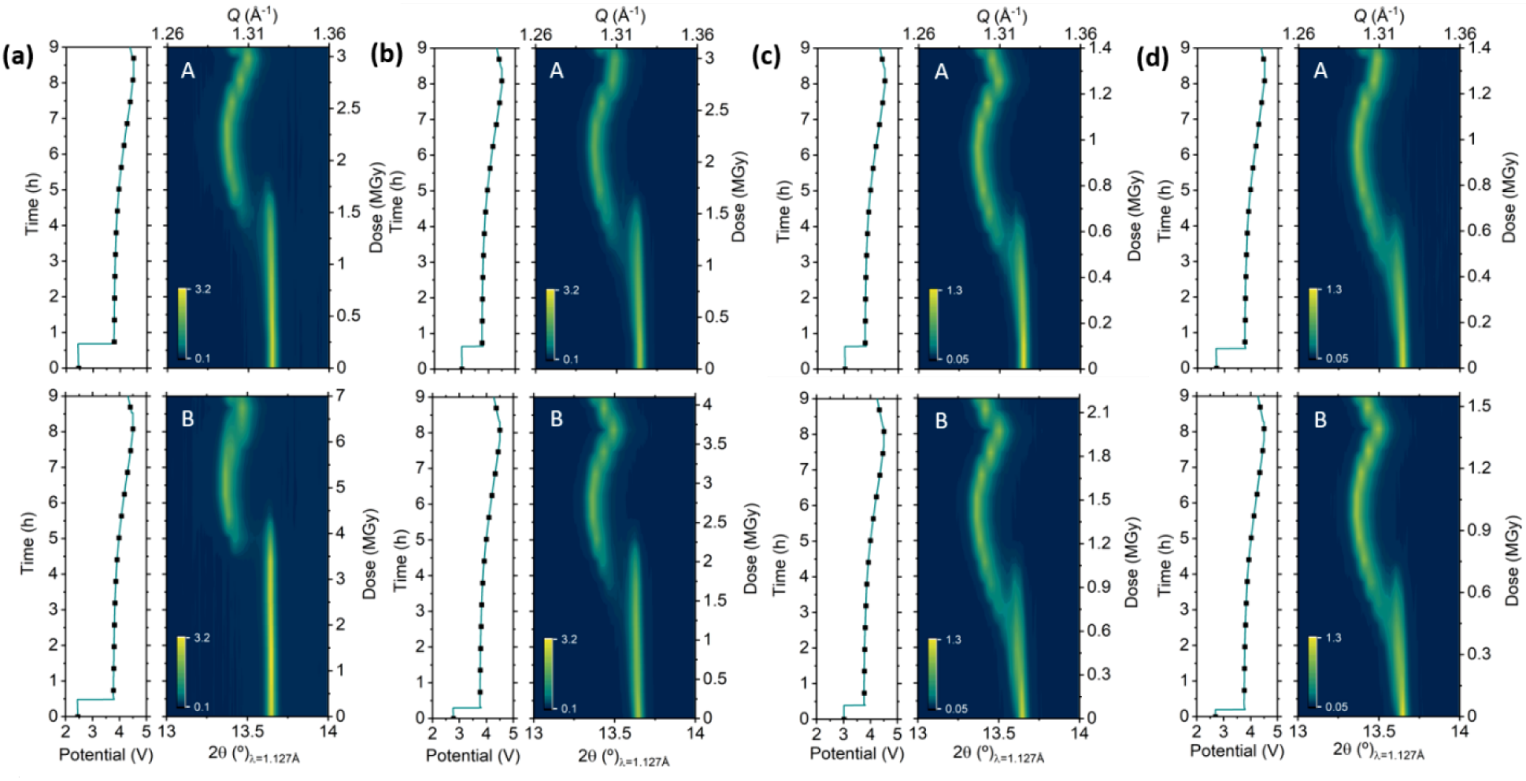
*Operando* X-ray diffraction patterns (zoom in on reflection 003) of NMC111 at spots A (up) and B (down) acquired at 11 keV with attenuation of 100 μm (a,b) and 250 μm (c,d) of Al, where XANES (b,d) or EXAFS (a,c) of K-edge of Ni was measured at points B and corresponding electrochemical curves. Dots marked in the latter indicate measurement points.

In the interest of finding a dose limit below which beam effects can be avoided, a set of experiments with variable photon flux and exposure time was performed in four identical NMC coin cells. Figure 5 depicts the contour plots of the NMC111 (003) reflection as a function of time and radiation dose corresponding to a combined PXRD and XAS *operando* experiment for which A and B spots were exposed to the beam for 1 min when PXRD was collected, and in addition, at spots B, only Ni K-edge was measured for all four cells. The flux level was controlled by placing 100 μm Al attenuators in cells (a) and (b) and 250 μm Al attenuators in cells (c) and (d), reducing the photon flux at the energy of Ni K-edge by 70% and 90%, respectively. Under these two attenuation conditions, the additional exposition to the beam at spots B was limited to the time required to acquire either a XANES (1 min; spots B in cells (b) and (d)) or EXAFS (3 min; spots B in cells (a) and (c)) spectrum. These results indicate a clear correlation between the radiation dose and the degree of inhibition. Spot B of cell (a), where EXAFS was collected with the higher photon flux, shows the strongest delay in the evolution of the diffraction pattern, while spot B of cell (d), where XANES was collected with a lower photon flux, shows that changes in the diffraction pattern are close to those expected. Experiments conducted with 100 μm Al attenuators (cells (a) and (b)) led to measurements with cumulative radiation dose exceeding 3 MGy/half cycle (dose rate exceeding 0.25 MGy/measurement with 23 min rest between consecutive measurements) with inhibition being still evident, while those performed with 250 μm Al allowed maintaining cumulative radiation dose below 2 MGy/half cycle (dose rate below 0.2 MGy/measurement with 23 min rest between consecutive measurements), and hence, the spectral evolution shows, if any, only small deviations from the expected behavior with no beam inhibition. No major differences in the degree of inhibition can be observed between spots A and B for cells (c) and (d), where the XANES and EXAFS measurements were acquired under strong attenuation levels, which resulted in a mere increase by 0.2 or 0.8 MGy/half cycle for spots B when compared to spots A. These results illustrate how the energy dependence of the photon flux at a beamline can dominate the contribution to the dose.

**Figure 6:**
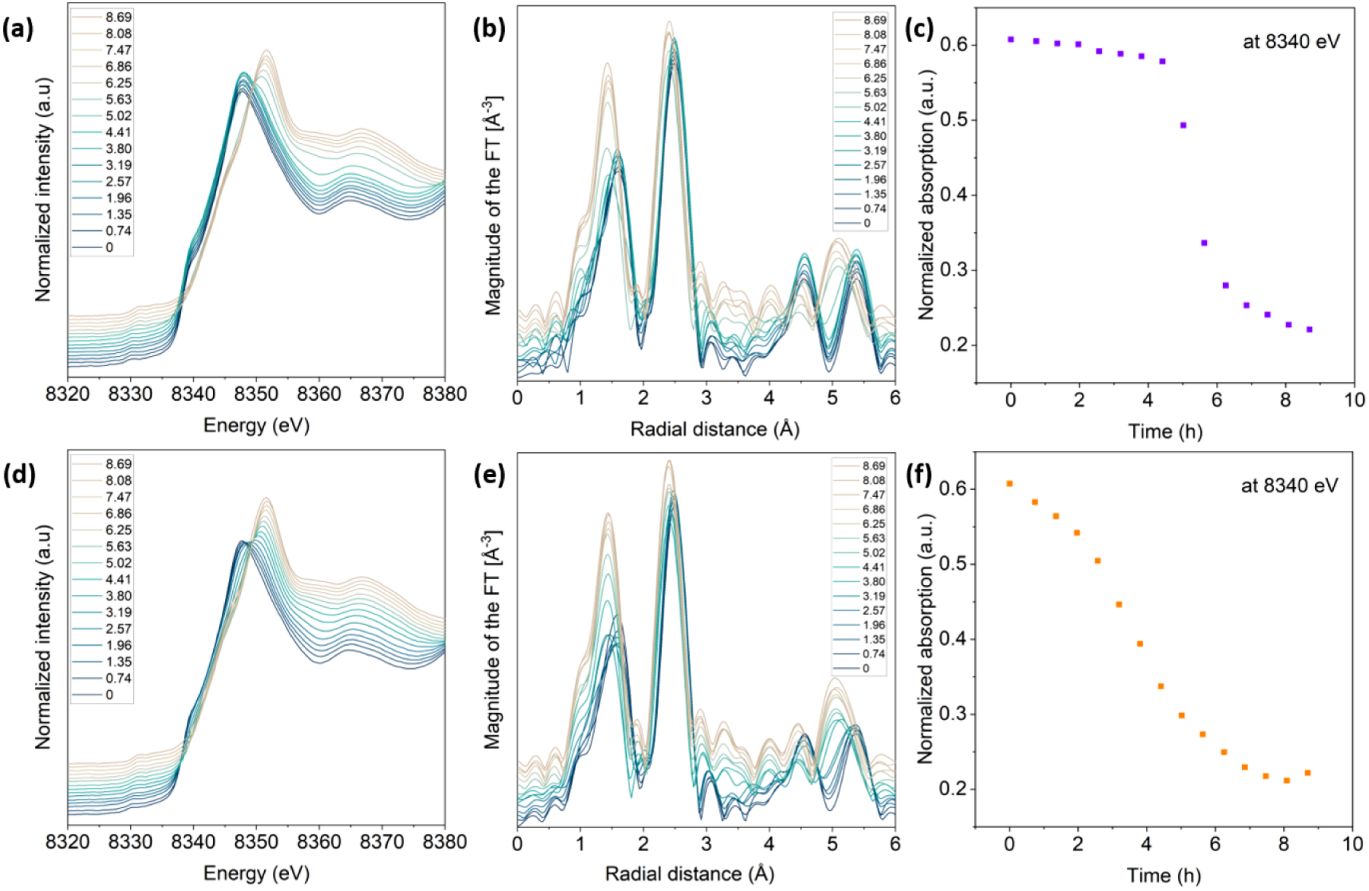
*Operando* EXAFS of Ni K-edge measured at B spots at attenuations levels of 70% (a) and 90% (d), their corresponding Fourier transform of the *k*^2^-weighted spectra (b) and (e), and selected energy cuts (c) and (f).

In order to showcase the correlation between the beam effects observed on the PXRD patterns with those present on the XAS spectra, Figure 6 illustrates the *operando* Ni K-edge XAS normalized spectra and the corresponding Fourier transform of the *k*^2^-weighted X-ray absorption fine structure (EXAFS) spectra for NMC111 electrodes measured at attenuation levels of 70% and 90% (Figure 4a,c, respectively). The spectra in Figure 6 depict a clear shift of the Ni K-edge to higher energy values upon charge, indicating Ni oxidation. The rising intensity at 8360 eV indicates the formation of Ni^4+^ species. The intense peak located around 1.3 Å is attributed to the closest oxygen shell (Ni–O), while the peak around 2.5 Å is assigned to the Ni–M shell (values not phase shift corrected).^35^ Strong changes in the first shell indicate that reversible changes in the oxidation state of Ni are accompanied by a shift to higher distance and a reduction in intensity (or vice versa). In a similar manner, as was observed in the PXRD, the variation of intensity of selected energy and *k*^2^-weighted EXAFS cuts depicted in Figure 6c,f showcase the partial hindrance induced in the cell measured with higher flux. This is reflected by an initial flat section, where the changes in the intensity of the rising band at 8360 eV are negligible for most of the oxidation followed by an abrupt jump when the cell is reaching the final stages of charge. The same behavior is also apparent in the intensity of k^2^-weighted EXAFS cuts corresponding to the first and second shells (Figure 6c). However, the equivalent features of the cell measured at 90% attenuation (Figure 6f) show a smooth reversible evolution of their intensities throughout cycling, indicating a low level of hindrance.

### 3.5 Beam Effects at High Energy

**Figure 7:**
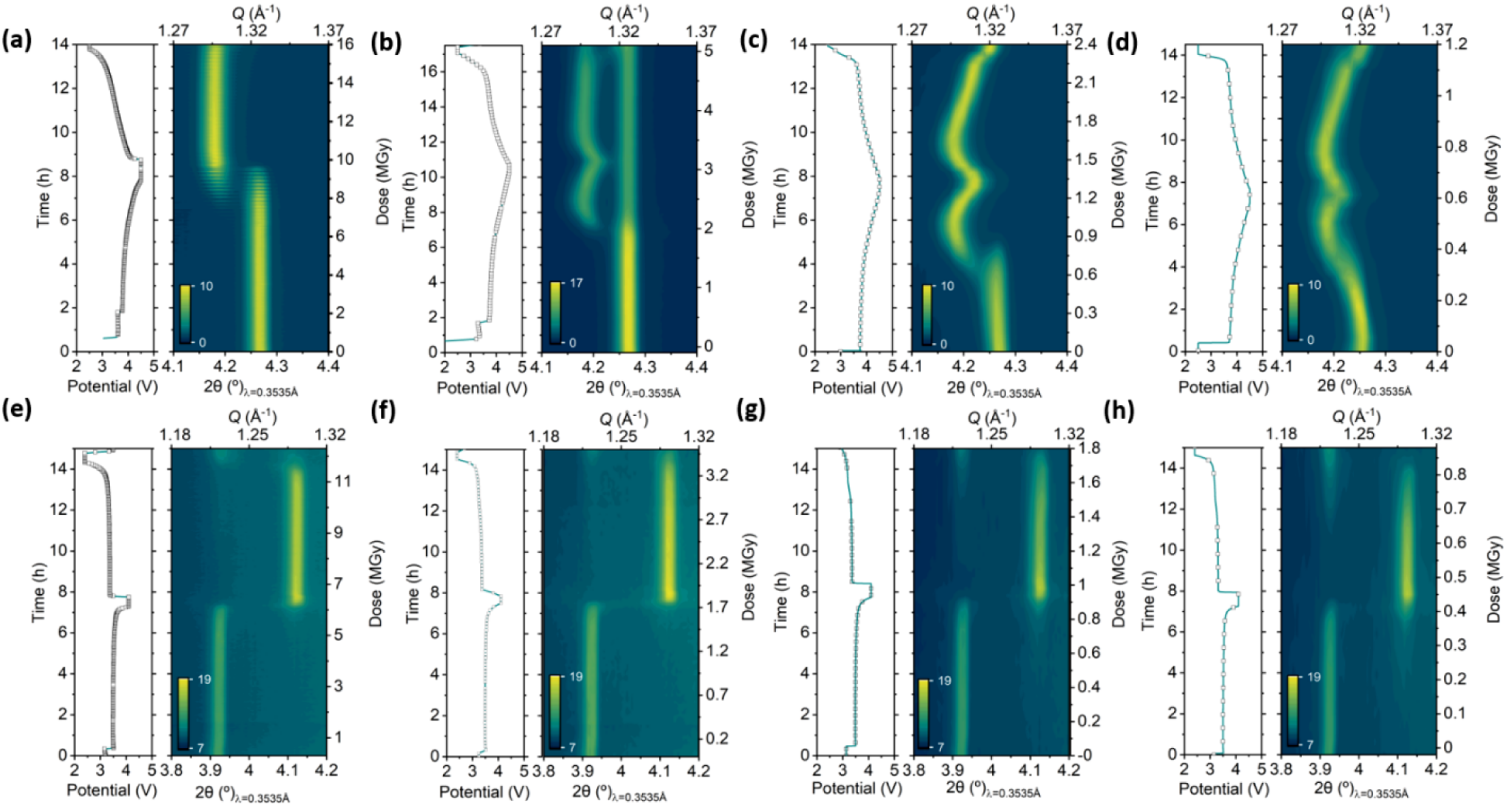
Zoom in on reflection (003) and (200) of *operando* X-ray diffraction patterns acquired using coin cells at 35 keV of NMC111 (a–d) and LFP (e–h), respectively, and corresponding electrochemical curves. Acquisition time was 27 s, and data were taken at 2.5, 10, 20, and 40 min intervals from (a–d) and (e–h). Dots marked in the electrochemical curve indicate measurement points.

The results described in previous sections suggest a potential influence of the radiation energy at which PXRD was acquired on the degree of beam inhibition induced. To further elucidate this potential correlation, a series of *operando* PXRD experiments were conducted at 35 keV at MSPD beamline. At this energy, much lower absorption is expected compared to previously employed 11 keV. Figure 7 shows the contour plots corresponding to the *operando* diffraction patterns of NMC and LFP collected following a measurement protocol that enables varying the dose rate (dose/(measurement + rest time)). NMC111 and LFP cells were cycled at C/10 and C/7, respectively. Pattern acquisition time was set to 27 s, and they were collected at 2.5, 10, 20, and 40 min intervals. The photon flux was estimated to be 5 × 10^11^ ph/s. Since the absorptions of the NMC and LFP electrodes are 0.05 and 0.02, the corresponding radiation doses were 0.05 and 0.032 MGy per pattern, respectively. NMC111 diffraction patterns collected every 2.5 min exhibit a significant deviation from the anticipated structural evolution; only negligible phase changes can be discerned from the PXRD (Figure 7a) throughout the entire oxidation step, which are followed by an abrupt evolution at the very end of the process. This pronounced inhibition persists during reduction. The diffraction patterns measured at 10 min intervals also exhibit notable deviations from the expected behavior with significant inhibition of reactivity below 4 V. A similar trend is also observed for experiments in which patterns were collected every 20 min. Only the experiment in which the largest time interval was set between measured patterns (40 min) showed minor inhibition, if any. Analogously, in the case of LFP, there is also a delay in the observation of the two-phase behavior. The phase transition is initiated only at the very end of each half cycle when the voltage changes fast. This effect is more pronounced for the samples measured at 2.5 and 10 min intervals, whereas the phase transition is slightly more evenly distributed throughout the cycle for the cells measured at 20 and 40 min intervals (see Figure 7g,h, respectively). Measurements with dose rates above 0.3 MGy/h with rests between measurements of 10 min or lower, which resulted in cumulative doses of 5 MGy/cycle or higher, result in pronounced inhibition effects, whereas the expected reaction mechanism is observable only for dose rates below 1 MGy/h with rest between measurements of 40 min (total doses below 1.2 MGy/cycle). Surprisingly, despite the reduced absorption at the tested energy, the total dose/cycle achieved under these measurement conditions remains in the order of a MGy to a few tenths of MGy, as the lower sample absorption is largely compensated by a higher number (flux) and energy of the incoming photon. The level of latency observed in these high-energy experiments is remarkably comparable to those observed in low-energy experiments described in previous sections, which exhibited similar total dose levels per cycle.

### 3.6 Influence of the Electrode Thickness

**Figure 8:**
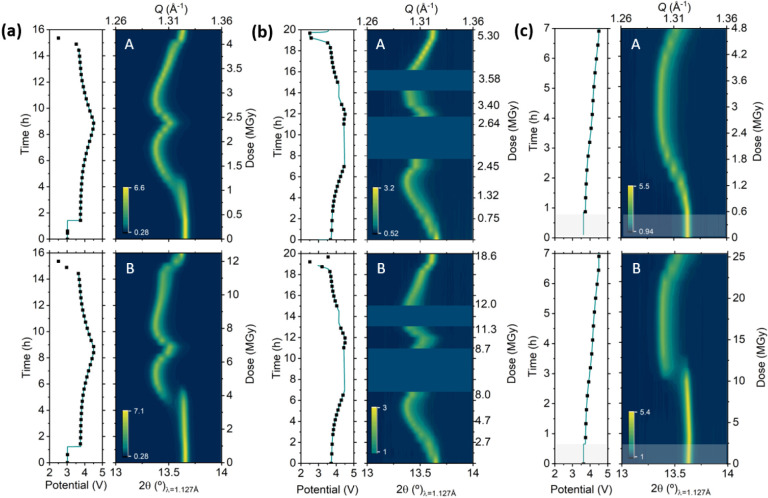
*Operando* X-ray diffraction patterns (zoom in on reflection 003) of NMC111 at spots A (up) and B (down) acquired at 11 keV in hexagonal LeRiChe’S v2 cells and corresponding electrochemical curves. Dots marked in the latter indicate measurement points. Active mass loading of 13.21 mg/cm^2^ and 100 μm Al attenuation was used for (a) and 2.92 mg/cm^2^ active mass loading for cells (b) and (c), with 100 μm Al attenuation or direct beam, respectively (dark blue stripes correspond to beam loss periods).

The experiments described above have been carried out with commercial electrodes with high active material loading, which often differs from the loadings of electrodes fabricated on a laboratory scale for fundamental research. Thus, and in order to assess the influence of the active material loading (i.e., thickness) in the effects discussed above, additional combined XAS and PXRD experiments were conducted at NOTOS beamline, following the same measurement protocol to compare typical NMC111 (13.21 mg/cm^2^ active mass loading and 70 μm thick), with a customized thinner NMC electrode also prepared by NEI corporation with a reduced active mass loading of 2.92 mg/cm^2^ and 17 μm thick. Two analogous experiments were conducted in LeRiChe’S v2 cells at two flux levels, using attenuation of 100 μm of Al and under direct beam (no attenuation). Figure 8a,b shows the contour plots of NMC 003 reflection for the thick and thin electrodes, respectively, both with the 100 μm Al attenuator. A significant delay in pattern evolution is evident for the thicker electrode, in particular for the measurements at spot B, while the thinner electrode, which has been equally exposed to the beam, shows almost negligible deviations from the expected behavior. Interestingly, these findings do not correlate with the estimated doses, as the absorbed photon flux decreased by a factor of 2.7 at the same time that the mass decreased by a factor of 4.5, resulting in an overall 1.7-fold increase in the dose for the thinner electrode compared to the thicker one. Given the low beam effects observed for the thinner electrode, a second cycle was conducted on the same cell removing all attenuators (Figure 8c), increasing the photon flux 1.7 fold at 11 keV with measurements being taken at new spots after prolonged rest time at open circuit potential. In agreement with the dose increase, strong beam inhibition effects are observed, particularly at spot B, where the long exposure times related to the Ni K-edge EXAFS measurement induced dose levels as high as 50 MGy/cycle. This exemplifies that the previously described critical dose dependency is also valid for thinner electrodes, despite the fact that its tolerable absolute threshold value is considerably higher.

### 3.7 Voltage Pulses Mitigating Effect

**Figure 9:**
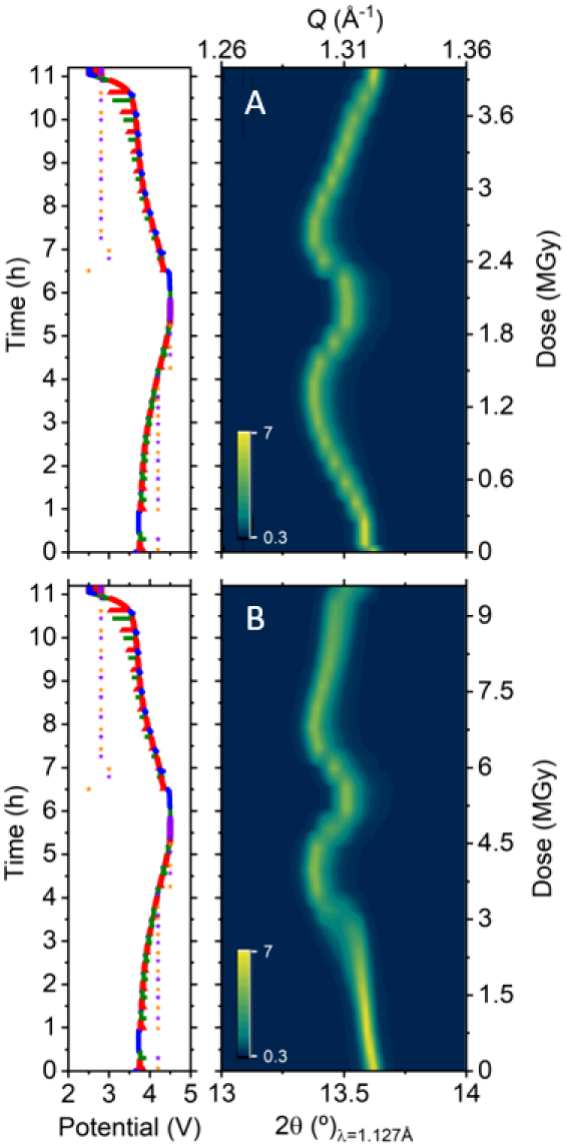
*Operando* X-ray diffraction patterns (zoom in on reflection 003) of NMC111 at spots A (up) and B (down) acquired at 11 keV in LeRiChe’S v2 cells and corresponding electrochemical curves. 100 μm Al attenuation was applied. Green and blue dots in the electrochemical curve correspond to the constant voltage pulses.

Several of the previously presented results on NMC111 and LFP electrodes repeatedly revealed that samples exhibiting notable beam-induced inhibition at the measurement spot would undergo abrupt phase transformations or partially recover the reactivity when the electrochemical curve experiences fast variations in voltage (see, e.g., Figures 1,6, and 7). Encouraged by this observation, we explored the effect of a cycling protocol that applies a short voltage pulse just after conducting each measurement. The objective was to explore if such pulses could have a mitigating effect while at the same time revealing additional features of the beam-induced reactivity inhibition mechanism. Figure 9 illustrates the contour plots of the (003) diffraction peak of a thick NMC111 electrode as a function of time and radiation dose along with the corresponding *V*–*t* curve. In these experiments, 1 min constant voltage pulses at 4.2 and 4.5 V upon oxidation and at 2.5 and 3 V upon reduction were applied immediately after each PXRD and XAS measurement. A visual comparison of the time-dependent evolution of the 003 reflection for both protocols (with and without pulses) can be inferred from Figures 9 and 8a. The evolution of the diffraction peak at spot A in the sample subjected to voltage pulses shows the expected behavior with no apparent beam inhibition effect, while at spot B, the voltage pulses seem to have had a mitigating effect, reducing the degree of hindrance.

## 4 Discussion

The results obtained from the series of *operando* experiments detailed above demonstrate how high-brilliance X-ray radiation can influence the electrochemical activity of the battery electrode active materials investigated at the measured spot. Similar effects have also been observed in *operando* PXRD experiments, when there was insufficient stack pressure within the cell resulting in inhomogeneous electrochemical reactivity of the electrodes.^44,45^ The intricate nature of these phenomena might blur the distinction between cell- and beam-derived effects, often leading to confusion or the conflation of one with the other. Therefore, the use of *operando* cells that ensure optimal stack pressure is essential to guarantee the acquisition of representative data. Once this is ensured, a clear correlation between the degree of hindrance and the radiation dose can be observed within electrodes of the same mass loading. The combined XAS and PXRD experiments conducted in Leriche 1.0, LeRiChe’S v2, and coin cells depicted in Figure 3 present comparable levels of inhibition at the sampled spot when exposed to similar dose rates. The radiation effect observed consists of a lack of reactivity at the sampled spot that can rank from a total reaction inhibition throughout the whole charge and discharge cycle (Figure 1) to a partial hindrance that can be partially recovered or to an almost negligible deviation from the expected mechanism (Figures 4 and 5), depending on the total dose and dose rate (see Figure S7 for a comparative plot of the evolution of Ni K-edge energy vs the percent of cell discharge capacity for several of the experiments reported above). These effects were consistently evident for both electrode materials and in both PXRD and XAS data (Figures 5 and 6) for experiments performed at several photon energies, in the vicinity and far above the absorption edge of the active elements (Figures 4 and 7), with different photon fluxes and diverse exposure times (Figure 5). The results obtained in this systematic study suggest that the total dose expressed in MGy/cycle consistently captures the magnitude of the beam-induced electrochemical delay at the measurement position, highlighting the relevance of the dose calculation as a reliable predicting tool to be used when designing *operando* experiments. In terms of total radiation dose per cycle, for the combined PXRD/XAS experiments where the resting time between acquisitions was fixed at 23 min and cells cycled between C/7 and C/10, spots irradiated with 70 MGy/cycle or more showed total inhibition of reactivity, spots with dose between 3 and 10 MGy/cycle presented from moderate to strong hindrance, and those below 3 MGy/cycle showed minor hindrance.

Data acquired at spots that had not been previously exposed to the beam during the *operando* experiment revealed the expected behavior (Figure 2). *Operando* measurements irradiating relatively close spots (3 mm) exhibited independent degrees of inhibition proportional to the received dose, pointing toward a highly localized phenomenon, whose spatial extension is constrained to the close vicinity of the irradiated spot. These findings align with those observed in μPXRD mapping, as reported by Christensen et al.^28^ The extent of the observed radiation effects appears to be limited to a inhibition of reactivity, as no other indications of structural or spectral changes were discerned neither in PXDR nor in XAS data. These reaction derogation phenomena are transient, as normal functioning of the affected area can be recovered, as highlighted in Section 3.4. Hence, we propose using the term “beam effect” instead of “beam damage” to more accurately describe the caducity of the observed phenomena.

The time interval between consecutive measurements required to avoid beam effects is significantly longer than the exposure time, which suggests that the electrochemically affected area remains inactive for extended periods of time (up to >20 min) after irradiation stops. This highlights the intricate nature of the beam-induced mechanism, involving effects that persist beyond the radiation duration but are reversible within a matter of minutes. The experiments that yielded results displaying evident signs of beam-induced hindrance (see Figures 1,3,7,8a, and 9) exhibit no discernible difference in the extent of the effect upon oxidation and reduction. In other words, hindrance seems to be independent of the sense of current flow, at least within the cycling rate and dose rate ratios examined. A consistent trend emerges when comparing multiple experiments, regardless of the material investigated (LFP and NMC111) and at different dose levels. The persistence of hindrance appears to be influenced by the voltage profile, with inhibition being more prominent when it is flat, particularly evident for LFP. In contrast, reactivity is recovered or partially restored toward the end of the charge and the beginning of discharge, where the voltage profile undergoes rapid changes (drastic increase in d*V*/d*t*, sloping *V*, vs time profile). This observation could, to some extent, explain why LFP, exhibiting a single constant voltage plateau upon operation, seems to be more vulnerable to radiation effects. These findings are in agreement with the mitigating effect observed when a voltage pulse is applied immediately after data acquisition (Figure 9). The relationship between voltage and the inhibition mechanism could be related to the induction of local internal resistances, which hence require an overpotential to recover electrochemical activity. The comparative study conducted on thick and thin electrodes revealed lower degrees of beam-induced hindered reactivity for the latter, contrary to what could be expected from the doses calculated. The latter primarily consider beam-dependent factors such as photon energy, flux, exposure time, and absorbed radiation, thereby overlooking the true complex nature of inhibition phenomena. This complexity arises from the interaction of radiation with the absorber embedded in a functioning electrochemical device undergoing charge and discharge cycling. Consequently, nonbeam-related factors, including electrode thickness and various physical and chemical properties of the electrode and electrolyte constituents, may also play a decisive role in the beam inhibition mechanism.

Several reasons may explain why thinner electrodes are less affected by irradiation. It could be linked to better thermal conductivity for thinner electrodes (higher current collector/mass ratio). The permeability of the electrolyte within thinner electrodes might facilitate quicker recovery after the irradiation period. Alternatively, lower ionization and the generation of fewer secondary electrons could also be factors, as photo and Auger electrons might have shorter dissipation paths, favoring their emission from the electrode.

One of the aspects to be considered to explain the inhibition mechanism is that the beam induces a local temperature increase sufficient to either directly affect the material or cause electrolyte solvent evaporation (and local drying). Blondeau et al.^27^ estimated an increase in temperature at the electrode and electrolyte of 2–3 °C based on beam power using finite element analysis (without considering thermal exchanges), in agreement with Bras et al. results.^14^ Such a beam-induced local temperature increase seems insufficient to cause a liquid–gas transition in the electrolyte; yet the possibility of this change in temperature playing a role in reaching a supersaturating state for the nucleation of gas bubbles cannot be dismissed. Schellenberger et al. found that the generation of gas bubbles in *in situ* soft XAS resulted from the convergence of radiolysis and thermal heating of the electrolyte, with Ar dissolved in the electrolyte, along with CO_2_ and CO resulting from electrolyte solvent decomposition, being the major constituents of the formed gas bubbles.^46^

**Figure 10:**
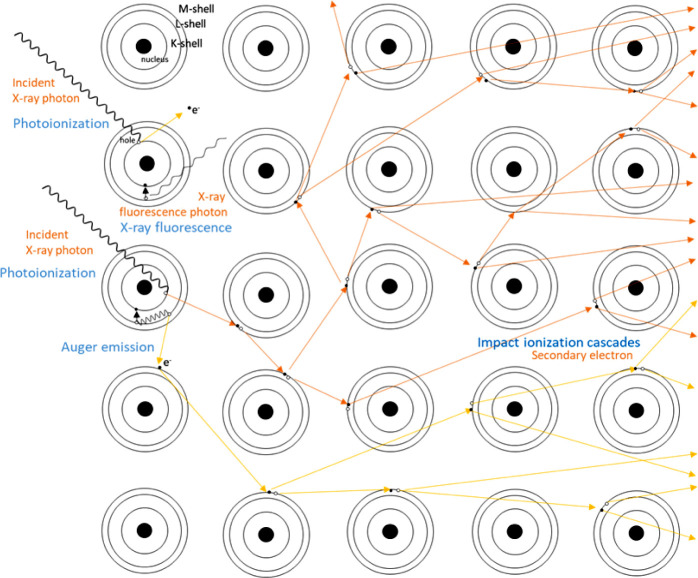
Illustration of the photoionization process that takes place after the absorption of an X-ray by an atom and the following ionization cascades and the generation of secondary electrons by inelastic scattering. Arrows represent the trajectories of the ejected secondary electrons generated from the fast photoelectrons (orange) and Auger electrons (yellow).

To better understand the underlying beam inhibition mechanism, it is crucial to revisit the intricate sequence of events triggered by the interaction between an X-ray photon and an absorber (Figure 10). Initially, a photoelectron is ejected, carrying away the photon’s energy minus the binding energy of the electron. This results in photoionization of the atom, leaving it in an excited state with two primary relaxation pathways. The first pathway involves X-ray fluorescence, where an outer-shell electron fills the core-hole created by photoabsorption, releasing a fluorescence photon with energy corresponding to the orbital energy difference. Then, the outer-shell hole further decays through a cascade of Auger processes. The second pathway is Auger decay, wherein an electron from the outer shell refills the core-hole, and the energy difference between outer- and inner-shell electron levels is carried away by an Auger electron (150–500 eV), leaving the atom doubly ionized. Both photoelectrons and Auger electrons have sufficient energy to ionize nearby atoms through direct collisions. The deeper in the bulk of the particle the photoionization process is initiated, the less probable it is that the photo and Auger electrons will reach the surface and leave the particle. Instead, they will undergo a relaxation process by inelastically scattering with the surrounding atoms, initiating an ionization cascade as they collide with other electrons. This transfers a fraction of their energy to those secondary electrons and causes a rapid increase in the ionization level of the atoms and the local temperature in the sample. An estimated 6 keV photoelectron leads to the creation of approximately 300 secondary electrons through impact ionization cascades before reaching the thermal equilibrium with the surrounding medium.^47^ Secondary electron energy remains photon-independent and typically below 20 eV with a few eV FWHM. It has been shown how the high degree of ionization and the secondary electrons that follow the photoionization are responsible for radiation damage and radiolysis under intense XFEL radiation in protein crystals.^25,26^ Radiation damage in TEM and SEM is also related to electron scattering processes^48^ and has been previously identified as one of the most relevant contributors to beam damage by several recently released phenomenological reports on beam effects in battery materials.^14,27,29^

**Figure 11:**
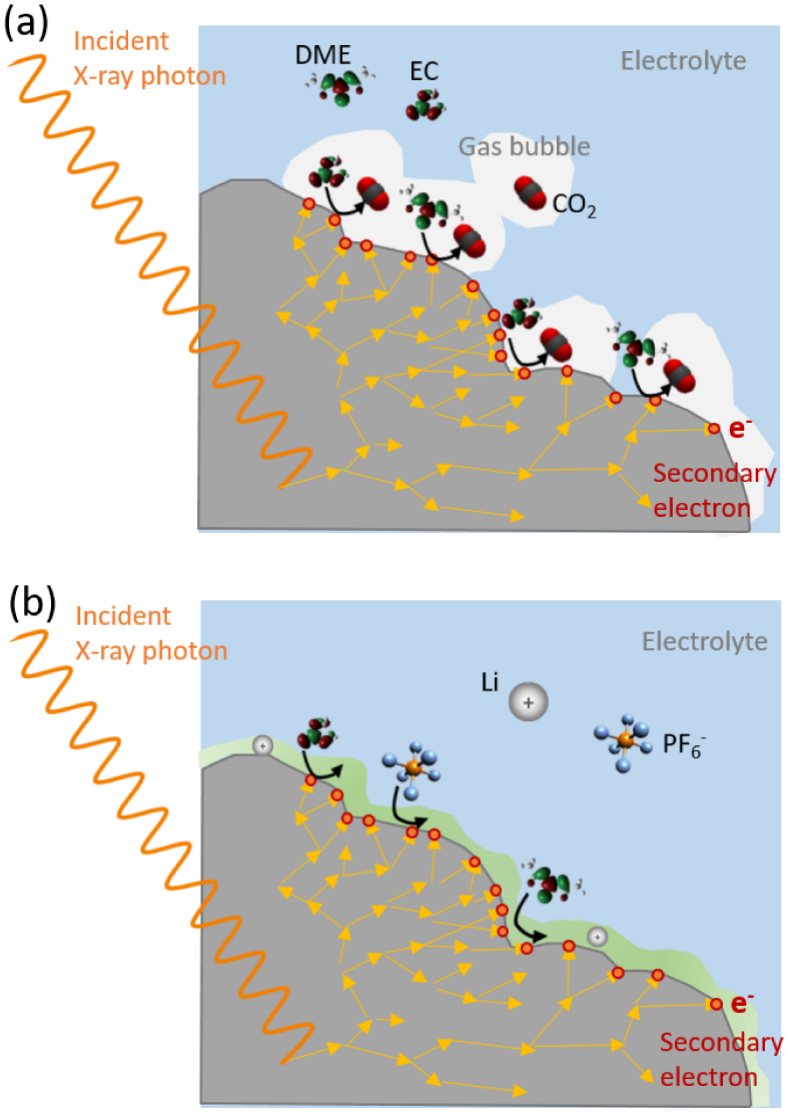
Illustration of the secondary electron cascade generated by inelastic scattering following the photoionization and two plausible interactions with the medium at the particle–electrolyte interface: (a) generation of gas bubbles and (b) formation of a passivating layer by decomposition of the electrolyte.

However, uncertainties persist regarding how the photoionized particles and secondary electrons will interact with the absorber environment within an electrochemical cell under *operando* conditions. In the first place, the irradiated particles are in an excited state in which they have accumulated a significant amount of charge carriers. It is unclear how long they can stay in this excited state; thermalization should take place in the millisecond range. However, this process may be highly dependent on the local conducting properties and the availability of recovering lost electrons from the drain. In a first scenario where the particles may remain in an electrostatic excited state for a prolonged period, it is uncertain whether they could establish some Coulombic interaction with the milieu that prevents normal ionic migration from the electrolyte to the charged particles. Given this assumption, thinner electrodes with improved conduction could facilitate the drain of charges, and drastic voltage changes might drive the discharge of the excited particles. A second conceivable scenario is that photoionized particles and secondary electrons reaching the surface induce radiolysis of the electrolyte, producing gases that nucleate and form small bubbles. These bubbles may become trapped on the particle surface or within the intricate pores of the electrode layer (Figure 11a). This mechanism aligns with the prolonged relaxation times required for irradiated zones to regain reactivity, potentially linked to the time necessary for the electrolyte to displace or redissolve the gas and adequately rewet the dried area. The behavior of the generated bubbles can be somewhat stochastic, offering a partial explanation for the abrupt changes in reactivity observed at the irradiated points. Nevertheless, the consistent and sudden reactivity shifts noted at the end of charge and discharge, particularly during rapid voltage changes, suggest a correlation with alterations in resistivity and the accumulation of overpotentials at the particle–electrolyte interface. Finally, in a third scenario, the highly ionized particles and secondary electrons could induce catalytic decomposition of solvents, salts, binder, and/or conductiving carbon at the particle interface, forming a thin, poorly ionically conducting layer of decomposition products (refer to Figure 11b). We might envisage that the partial polymerization of the solvent can form a jelly zone surrounding the particles, where the electrolyte loses its ion conducting properties, increasing resistance at the interface. In the presence of the local electric field induced during photoionization, a high concentration of ionic species might accumulate at the interface, potentially contributing to the inclusion of ionic species into this decomposition layer, which might affect its solubility and overall stability. The latter could explain the voltage-dependent inhibition, as the strength of the hindrance and the overpotential needed to recover the reactivity could be proportional to the thickness of the passivating layer, while the recovery of reactivity during periods of rest in the absence of irradiation might be associated with its gradual dissolution.

In conclusion, the arguments discussed above point toward the photoionization and generation of secondary electrons reaching the particle–electrolyte interface being the primary drivers of beam effects. The number of secondary electrons generated depends not only on the incoming radiation but also on the material nature and its particle size, as photoelectrons generated near the surface will leave the particle, while those generated deep within the bulk will thermalize before reaching the surface. Furthermore, these hypotheses enable us to explain discrepancies encountered in dose calculations for both thick and thin electrodes. The observed hindrance is not solely dependent on the secondary electrons reaching the surface but might also depend on the ionic conductivity of the layer of electrolyte surrounding the particles, which can be affected by the beam and the availability of fresh electrolyte. This renders the effect not only radiation-dependent but also highly contingent on the physical and chemical compositions of the electrode and electrolyte. Therefore, the probability of encountering beam effects during an *operando* experiment is difficult to predict.

The present study employed well-known materials under conventional operation conditions, facilitating the detection of biases in the results stemming from beam effects. However, *operando* experiments at synchrotron facilities typically involve novel materials or conventional ones subjected to extreme conditions. In these scenarios, the possibility of encountering beam-induced effects that escape detection is significant, potentially resulting in data misinterpretation. Therefore, this work aims to raise awareness of this problem among the *operando* battery community and identifies key aspects that can be used to detect, minimize, and prevent beam detrimental effects. First, estimating the dose before an experiment can help in assessing their likelihood. As a general guideline, beam effects should not be foreseen for total doses below 1MGy/cycle, while doses exceeding 50 MGy/cycle are prone to induce severe effects. For doses between 3 and 20 MGy/cycle, the occurrence of beam effects is uncertain. In such cases, factors like the rest time between measurements (dose rate) or nonradiation-dependent contributors such as electrode thickness or electrolyte properties might influence the appearance of beam-induced reaction inhibition. The dose equation proves valuable not only for estimating potential effects but also for developing effective mitigation strategies. For instance, modifying the spot size from a focused beam (0.3 × 0.5 mm) to a defocused beam (1 × 3 mm) would induce a notable 20-fold reduction in dose while conserving the photon flux and additionally enabling to average a larger region of the sampled material. Furthermore, using attenuators, reducing acquisition time, rest time between consecutive measurements, and changing energy (in PXRD experiments) are strategies to consider. It is also advisable to measure at multiple spots and establish a series of control points throughout the *operando* measurement, where data are acquired at a nonirradiated location to rule out the presence of beam effects and validate the reliability of the obtained data.

Finally, understanding the effects of radiation can guide developers in designing new machines and beamlines, particularly during synchrotron upgrades aiming for higher brilliance. For instance, ultrafast machines and detectors that enable data acquisition in seconds could potentially minimize the radiation dose. The observed radiation effects in synchrotron experiments exhibit similarities to those observed in studies on aerospace batteries designed to withstand high levels of cosmic radiation.^49−51^

## 5 Conclusions

The investigation of beam-induced effects on battery electrode materials under *operando* conditions conducted on well-known materials under conventional conditions has shed light on potential biases introduced by beam effects, demonstrating the significant influence of X-ray radiation on the electrochemical activity of battery electrode materials. The observed effects, ranging from total reaction inhibition to partial hindrance and negligible deviations from the expected mechanism, have been consistently observed across various experimental conditions, photon energies, photon fluxes, and exposure times. The total radiation dose per cycle has proven to be a useful predictor of the magnitude of beam-induced electrochemical delay when considering variations in all radiation parameters investigated. In this regard, we have evaluated dose ranges of likelihood of beam-induced effects that can serve as a guideline for the user community. However, the dose per cycle inadequately anticipated the level of hindrance when evaluating electrodes with varying mass loadings, emphasizing the complexity arising from the interaction of radiation with the operating electrochemical cell during operation. Nonbeam-related factors, such as electrode thickness and possibly other electrode and electrolyte properties, may play a critical role in the beam inhibition mechanism, which is still a matter of debate. Moreover, it may possibly have multiple origins that can contribute differently depending on the precise experimental conditions and material under investigation. The results obtained in this study indicate that photoionization and the generation of secondary electrons at the particle–electrolyte interface may trigger the catalytic degradation of the electrolyte and generate a poorly conducting decomposition layer, which could provide a plausible explanation for the transient nature of the beam effect as well as for the observed discrepancies in dose calculations for thick and thin electrodes. Indeed the effect is not solely radiation-dependent but contingent on the physical and chemical compositions of the electrode and electrolyte. The results presented herein also call for further research on radiation effects on batteries, which, aside useful in considering beamline designs and upgrades, could also be of interest to the aerospace industry.

## Supporting Information Available

Additional information on the *operando* cell setups and measurement protocols used in this study, complementary *operando* X-ray diffraction patterns, and absorption spectroscopy spectra, details on the dose estimation method and dose values per measurement type, and energy-dependent photon flux at NOTOS beamline in the configuration used for the experiments reported (PDF)
