# Supplementary material for: Beam Effects in Synchrotron Radiation Operando Characterization of Battery Materials: X-Ray Diffraction and Absorption Study of LiNi0.33Mn0.33Co0.33O2 and LiFePO4 Electrodes
Source: Chem Mater. 2024 May 17;36(11):5596–610. doi: 10.1021/acs.chemmater.4c00597 (PMC11170951; doi:10.1021/acs.chemmater.4c00597)
Supplement: Supplementary file 1 — cm4c00597_si_001.pdf [file cm4c00597_si_001.pdf]

Supplementary Information for

Beam effects in synchrotron radiation operando characterization of battery materials: X-ray diffraction and absorption study of $LiNi_{0.33}Mn_{0.33}Co_{0.33}O_2$ and $LiFePO_4$ electrodes

Ashley P. Black[a], Carlos Escudero[b], François Fauth[b], Marcus Fehse[c], Giovanni Agostini[b], Marine Reynaud[c], Raphaelle G. Houdeville[a], Dimitrios Chatzogiannakis[a,c], Joseba Orive[c], Alejandro Ramo-Irurre[a], Montse Casas-Cabanas[c,d*], M. Rosa Palacin[a*]*

[*]*corresponding authors:*

ablack@icmab.es

rosa.palacin@icmab.es

mcasas@cicenergigune.com

[a]Institut de Ciència de Materials de Barcelona, ICMAB-CSIC, Campus de la UAB, Bellaterra, 08193, Spain

[b]ALBA Synchrotron Light Source, Cerdanyola del Vallès, E-08290, Spain.

[c]Centre for Cooperative Research on Alternative Energies (CIC energiGUNE), Basque Research and Technology Alliance (BRTA), Alava Technology Park, Albert Einstein 48, Vitoria-Gasteiz, 01510, Spain.

[d]IKERBASQUE, Basque Foundation for Science, Plaza Euskadi 5, 48009 Bilbao, Spain.

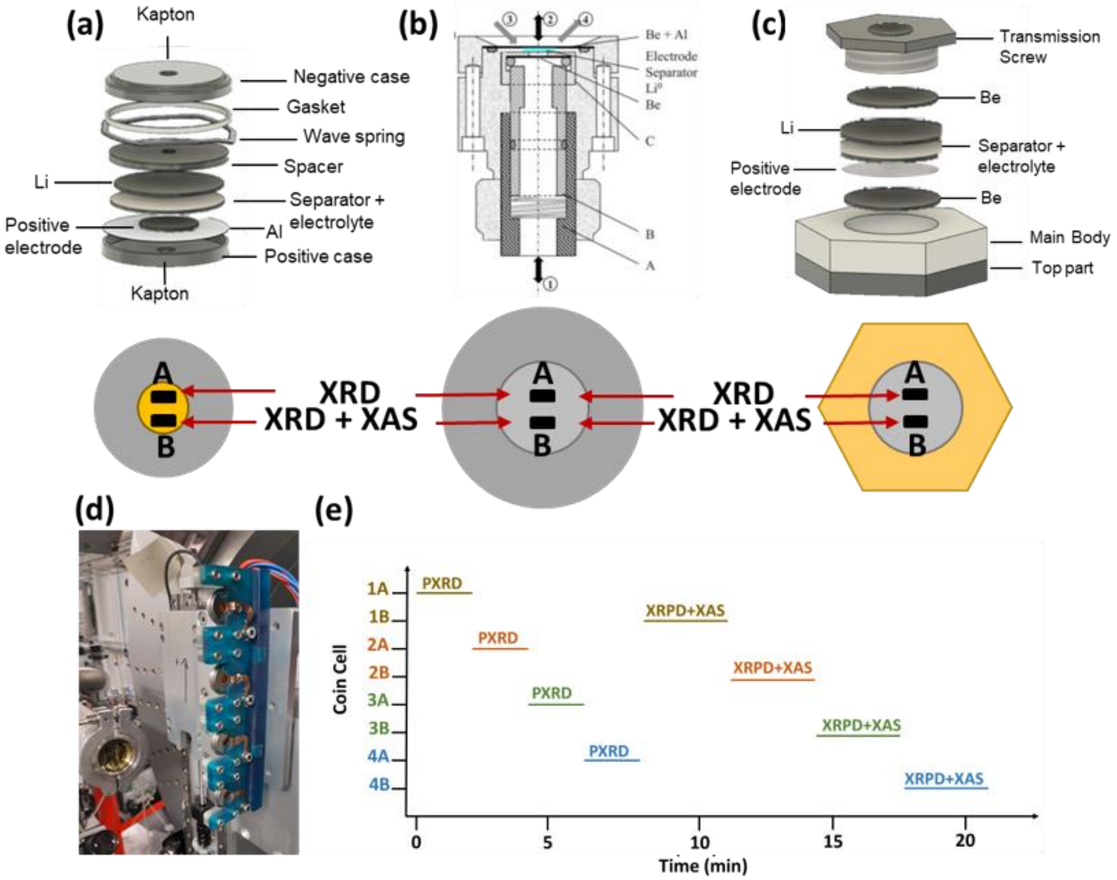

Figure S1. *Schematic layout, zenithal view and image of the setup of the operando cells Kapton window coin cell (a), Leriche 1.0 (b) and LeRiChe'S V2 (c). View of the carrousel with 4 coin cells mounted on the sample stage at NOTOS beamline (d) and a schematic representation of the protocol followed during combined XRD and XAS experiments involving measurements at two different spots.*

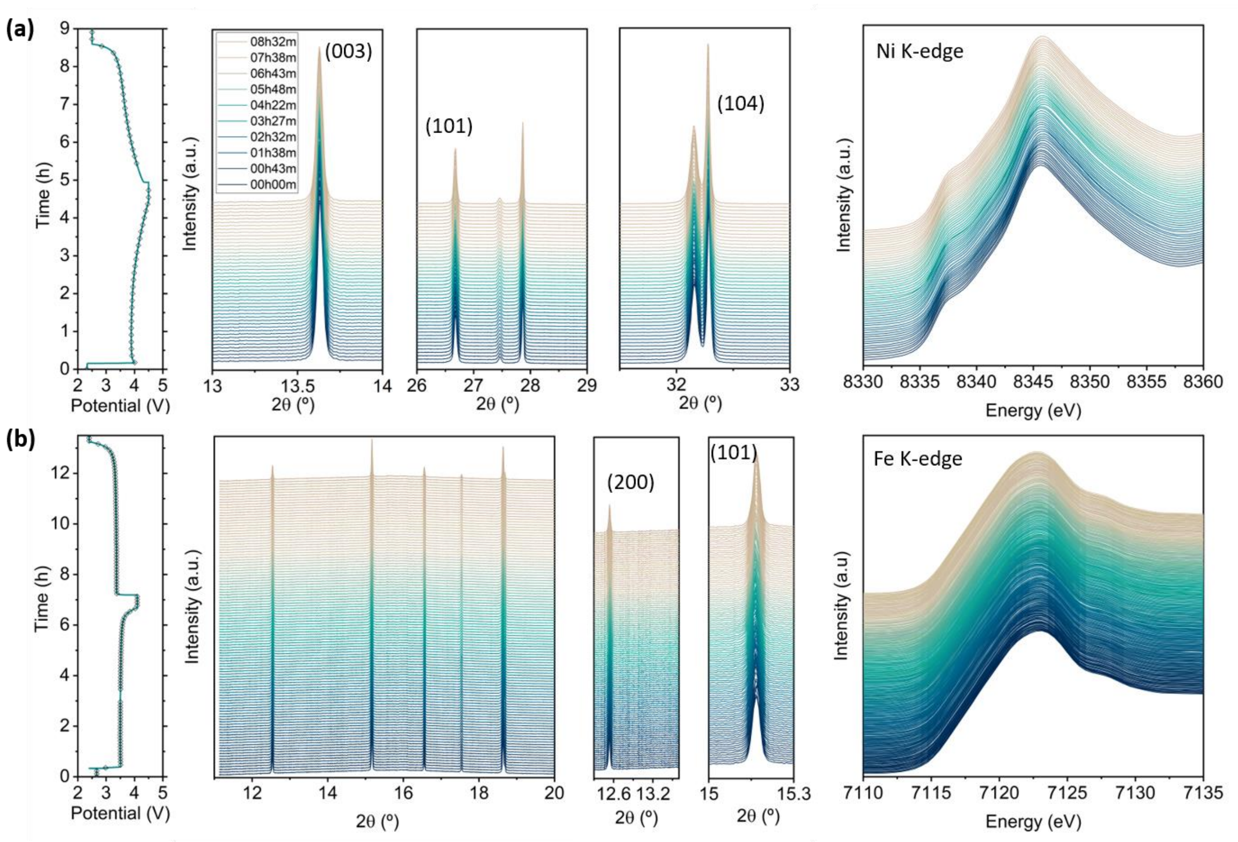

Figure S2. *Operando X-ray diffraction patterns (center), X-ray absorption spectra (right) and electrochemical curve (left) of NMC111 (a) and LFP (b) electrodes measured in Kapton window coin cells. (The cells were continuously exposed to the synchrotron X-ray beam for the sequential acquisition of the XRD patterns and XAS spectra).*

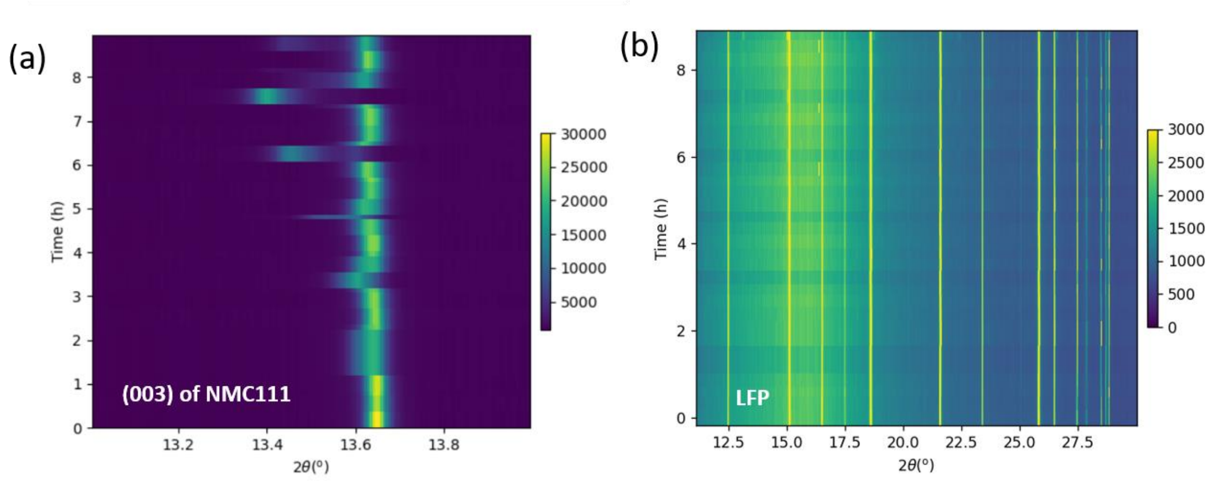

Figure S3. *Operando X-ray diffraction patterns (zoom in on reflection 003) of NMC111 (a) and LFP (b) acquired using Leriche* operando *cells with Be window corresponding to the first charge cycled in galvanostatic mode at C/10 rate. Measurements were conducted under continuous synchrotron light exposition at 5 spots in the cell. At four positions both PXRD and EXAFS of (Mn, Co, Ni) and Fe were performed for NMC and LFP, respectively. Due to small distance between points and error in measurement macro dose per position can't be reliably assessed.*

Dose estimation:

The dose as defined in equation (1) is specific for each electrode material and cells configuration and depends on the photon flux (F), Photon energy (E_{photon}), exposure time (t), transmittance at a given energy (T), the beam spot size (A), the electrode thickness (e) and density (d).

$$Dose\ (Gy) = \int_{E_i}^{E_f} \frac{F \times E_{photon} \times t \times (1 - T)}{A \times e \times d} \qquad (1)$$

In order to calculate the photon flux measurements with a 10 µm thick Si photodiode of 10 x 10 mm^2 area were taken at the sample position using the same beamline configuration as for the coin cells measurements (including or not the different attenuators). In this way, the flux attenuation due to all the different elements along the beamline was considered. Furthermore, all the elements present in the coin cell prior to reaching the electrode were taken into account. The transmission of 75 µm of Kapton or 200 µm of Be from the cell window was calculated from XOP software. In the same way, the transmission through the different attenuators (100 µm or 250 µm Al foil) used in the beam optics path, prior to reach the sample, was also calculated using XOP and was compared with the direct measurements using the Si photodiode with and without those filters, validating the XOP calculations. XOP was also used to estimate the attenuation related to the absorption of the separator and the electrolyte. In this case, the transmission of 420 µm of SiO_2 with 50% porosity with half of the thickness filled with electrolyte (210 µm) was considered to represent and the absorption of the electrolyte was estimated from its formulation. The transmission of the active material was calculated with XOP software based on the electrode composition and porosities specified by electrode supplier (NEI corporation).

Table S1. *Calculated X-ray radiation dose per measurement*

Cell type	Electrode material	Energy XRPD (eV)	Element K-edge XAS	Energy range XAS	Acquisition Time (s)	Attenuation (µm Al)	Dose (MGy)
Coin cell	NMC111	11 000	-	-	60	-	0.3402
Coin cell	NMC111	11 000	-	-	60	100	0.1991
Coin cell	NMC111	11 000	-	-	60	250	0.0892
Coin cell	NMC111	11 000	-	-	240	100	0.7965
Coin cell	NMC111	11 000	-	-	240	250	0.3567
Coin cell	NMC111	35000	-	-	27	-	0.0110
Coin cell	NMC111	-	Mn	6510- 6643	60	100	0.0036
Coin cell	NMC111	-	Mn	6510-6643	60	250	0.0001
Coin cell	NMC111	-	Mn	6480-6680	72	-	0.0485
Coin cell	NMC111	-	Co	7645-7845	60	100	0.0300
Coin cell	NMC111	-	Co	7645-7845	60	250	0.0032
Coin cell	NMC111	-	Co	7630-8330	210	-	0.5434
Coin cell	NMC111	-	Ni	8280-8480	60	100	0.0628
Coin cell	NMC111	-	Ni	8280-8480	60	250	0.0106
Coin cell	NMC111	-	Ni	8240-9140	60	100	0.2482
Coin cell	NMC111	-	Ni	8240-9140	60	250	0.0518
Coin cell	NMC111	-	Ni	8200-9000	192	-	0.7306
Coin cell	LFP	11 000	-	-	60	-	0.2974
Coin cell	LFP	11 000	-	-	60	100	0.1741
Coin cell	LFP	11 000	-	-	60	250	0.0780
Coin cell	LFP	11 000	-	-	240	100	0.6964
Coin cell	LFP	11 000	-	-	240	250	0.3119
Coin cell	LFP	35000	-	-	27	-	0.0071
Coin cell	LFP	-	Fe	7085-7250	60	100	0.0181
Coin cell	LFP	-	Fe	7085-7250	60	250	0.0011
Coin cell	LFP	-	Fe	7010-7710	140	-	0.3302
Leriche 1.0	NMC111	11 000	-	-	60	100	0.2013
Leriche 1.0	NMC111	-	Mn	6480-6680	72	100	0.0048
Leriche 1.0	NMC111	-	Co	7630-8330	210	100	0.1451
Leriche 1.0	NMC111	-	Ni	8200-9000	192	100	0.2522
Leriche 1.0	LFP	11 000	-	-	60	100	0.1760
Leriche 1.0	LFP	-	Fe	7010-7910	324	100	0.0739
Leriche 2.0	NMC111	11000	-	-	38	100	0.1275
Leriche 2.0	NMC111	-	Ni	8240-9140	180	100	0.2549
Leriche 2.0	NMC111 Low	11000	-	-	38	-	0.3219
Leriche 2.0	NMC111 Low	11000	-	-	38	100	0.1884
Leriche 2.0	NMC111 Low	-	Ni	8240-9140	180	-	1.3739
Leriche 2.0	NMC111 Low	-	Ni	8240-9140	180	100	0.4774

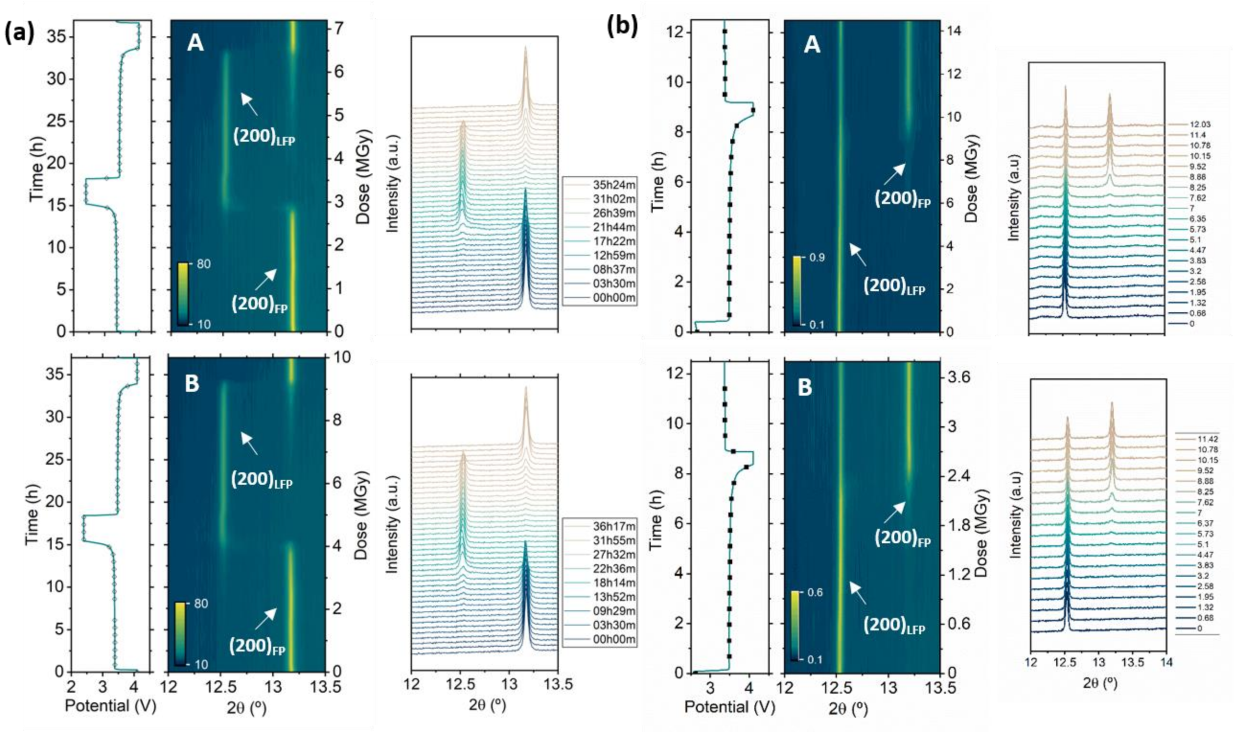

Figure S4. *Operando X-ray diffraction patterns (zoom in on reflection 200) of LFP at spots A (up) and B (down) and corresponding electrochemical curve acquired in similar conditions in Leriche 1.0 cells cycled at C/15 (a) and in Kapton window coin cells cycled at C/7 (b). Spot B in cell (b) was exposed to the beam for 3 min after each 1 min PRDX at 11 keV. Cell (a) starts in discharge due to error in macro during the first charge, then the cell was kept at constant voltage (4.5 V) off the beam for 3h prior to discharge and measurement was conducted at a fresh spot. Dots marked in the electrochemical curve indicate measurement points.*

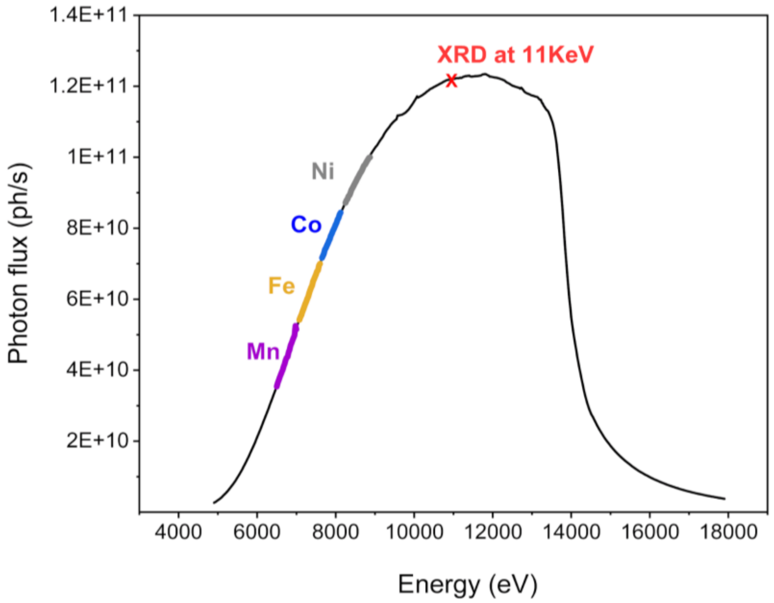

Figure S5. *Photon flux at NOTOS beamline at the sample position in the beamline configuration used for these experiments.*

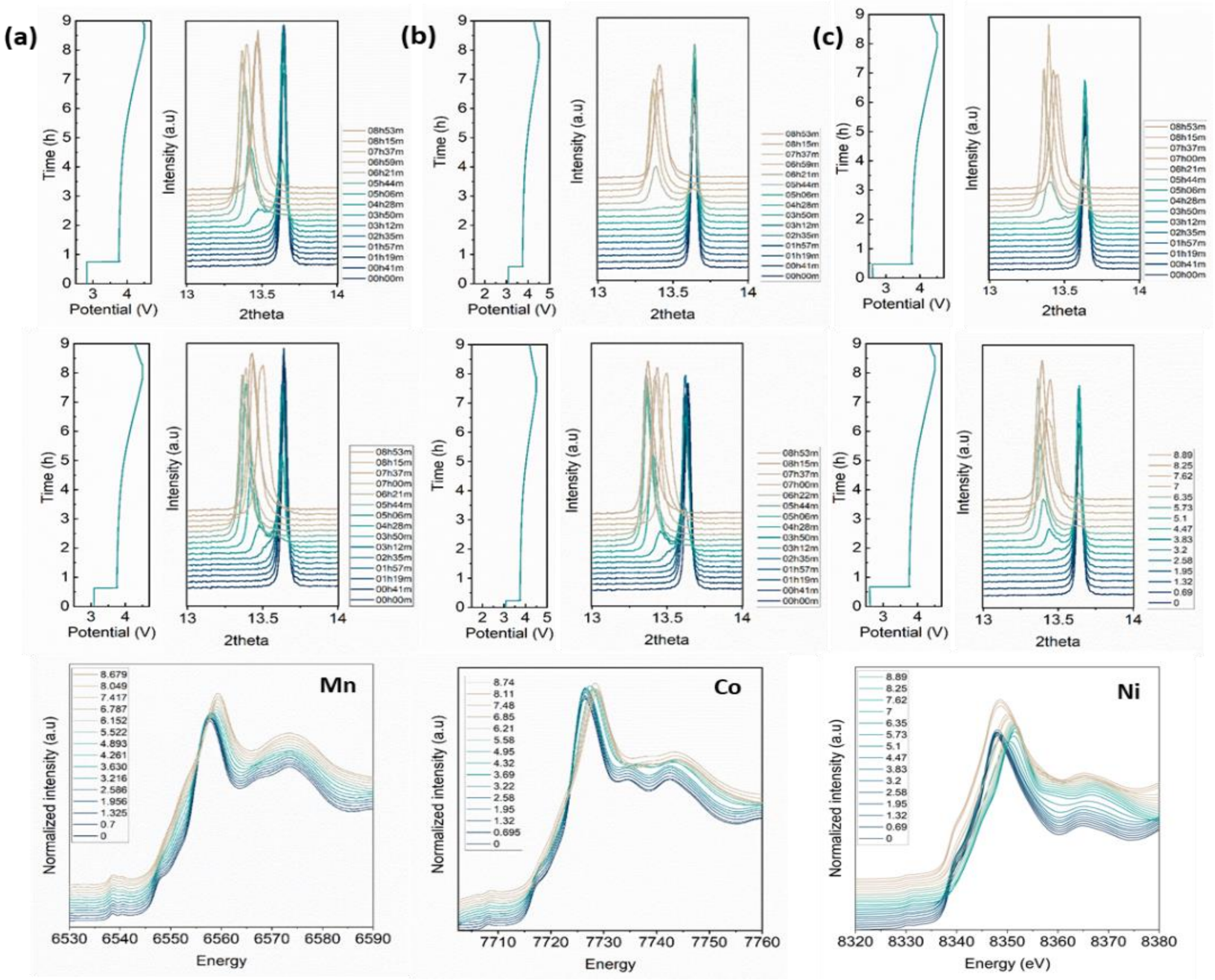

Figure S6. *Operando X-ray diffraction patterns (zoom in on reflection 003) of NMC111 at spots A (up) and B (center), together with the corresponding X-ray absorption spectra measured only at spots B (down) and their corresponding electrochemical curve acquired using Kapton window coin cells under different measurement conditions. Exposure time to 11 KeV radiation was 4 times longer per measurement in point A of cell (b) than for cells (a) and (c). At point B only K-edge spectra of Mn, Co and Ni were respectively measured on cells (a), (b) and (c).*

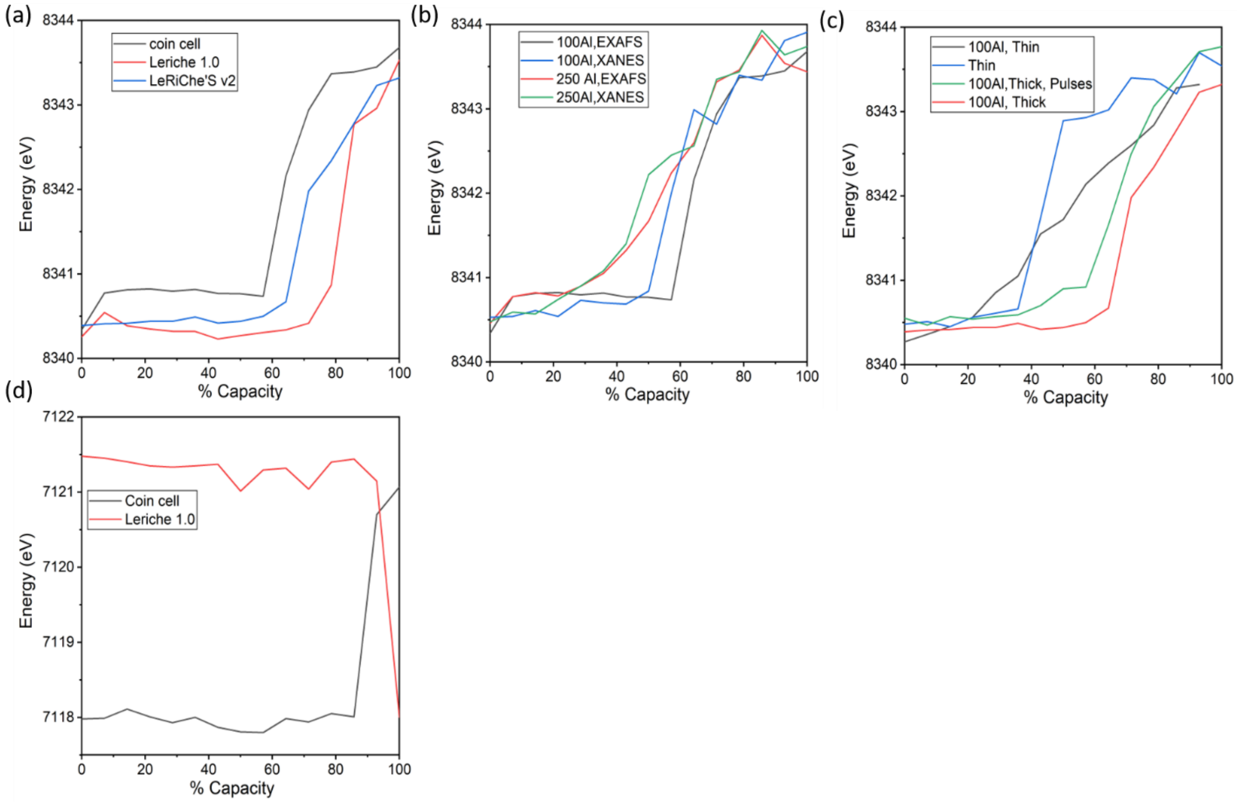

Figure S7. *Ni K-edge energy vs. % of charge capacity for NMC111 electrodes tested: in operando cells with different geometry (a), in coin cells under diverse photon flux and exposure times (b), and in LeRiChe´S v2 cell with different electrode thicknesses and with a cycling protocol involving voltage pulses (c).*
